# Watching eyes effect: the impact of imagined eyes on prosocial behavior and satisfactions in the dictator game

**DOI:** 10.3389/fpsyg.2023.1292232

**Published:** 2024-01-10

**Authors:** Jieyu Lv, Yuxin Shen, Zheng Huang, Chujian Zhang, Jiangcheng Meijiu, Hongchuan Zhang

**Affiliations:** Department of Psychology, Central University of Finance and Economics, Beijing, China

**Keywords:** altruistic behavior, watching eyes effect, imagined eyes, egoistic norm, dictator game with communication

## Abstract

The concept of the watching eyes effect suggests that the presence of eye or eye-like cues can influence individual altruistic behavior. However, few studies have investigated the effects of imagined eyes on altruistic behaviors and the psychological measures of dictators and recipients in the dictator game. This study used a 2 (Presentation Mode: Imagined/Visual) 2 (Cue Type: Eye/Flower) between-subject design and measured the effects of recipients' psychological variables and the communication texts between the dictator and the recipient. The results showed that there was a significant interaction between Presentation Mode and Cue Type. In the imagined condition, the dictator exhibited more altruistic behavior than in the visual condition. However, there was no significant difference in altruistic behavior between the Imagined Eye and Imagined Flower conditions. In addition, the study found that the Cue Type had a significant main effect on the recipients' satisfaction with the allocation outcome. Notably, in the Visual Flower condition, the dictator used more egoistic norm words when communicating with the recipient than other conditions. This study provides novel evidence on the effect of imagined social cues on individual behavior in the dictator game, and to some extent validates the robustness of the watching eyes effect under manipulation of higher-level verbal cognitive processes. At the same time, the study is the first to explore the impacts on recipients' psychological variables and the communication texts. These efforts offer new insights into the psychological and cognitive mechanisms underlying the watching eyes effect.

## 1 Introduction

Altruistic behavior, a form of prosocial behavior, refers to actions that are costly to the actor yet benefit others (Hamilton, 1964; West et al., 2011). It occurs within kins, friends, strangers and even animals. Altruistic behaviors promote life meaningfulness for individuals, coherence for group, and stability for organizations (Xie et al., 2017). Altruistic behavior is motivated by altruism or egoism. Empathy-induced altruism, reputation for future benefits, punishment avoidance by against rule are more likely to induce altruistic behavior. Except empathy-induced altruism, reputation or punishment avoidance are influenced by social presentation cues. When others present, reputation management and punishment avoidance are more likely to activate. Therefore, social presentation cues (e.g., eye) lead individuals behave more norm-obey or reputation-enhance behaviors.

Watching eyes effect refers to the phenomenon that presenting eye or eye-like figures altered individuals' behavior (Wu and Cui, 2020). The presentation of eye cues changed individuals' behaviors, for example, more prosocial behavior (Wang et al., 2011; Baillon et al., 2013; Sparks and Barclay, 2013), less immoral behavior (Nettle et al., 2012), and less antisocial behavior (Dear et al., 2019). Eye cues not only affected behaviors observed in laboratory, e.g, trust (Xin et al., 2016), dishonest (Cai et al., 2015), altruistic behavior (Rigdon et al., 2009), and cooperation (Burnham and Hare, 2007), but also affected behaviors in the field, e.g., blood donation (Senemeaud et al., 2017), litter recycling (Francey and Bergmüller, 2012), and littering (Bateson et al., 2013). However, recent studies have failed to replicate the watching eyes effect (Tane and Takezawa, 2011; Rotella et al., 2021). Similarly, some meta-analyses have reported varying results, suggesting that eye cues do not influence the likelihood of generous donations or the average donation amount (Northover et al., 2017a), have an effect size near zero on moral judgments (Northover et al., 2017b), but can significantly reduce the frequency of antisocial behavior (Dear et al., 2019). However, Oda (2019) criticized these null findings, arguing that previous meta-analyses did not control for moderating factors. A more recent and inclusive meta-analysis (Wang et al., 2023), which more comprehensively integrated existing studies on the watching eyes effect and included some moderating variables, reported a small but significant impact of eye cues on pro-social behavior. Despite researchers' in-depth and varied explorations into the phenomena and mechanisms of the watching eyes effect, it continues to be a subject of controversy. One of the novelty of this study lies in re-validating the robustness of the watching eyes effect in altruistic behavior. Therefore, we propose research Hypothesis 1a: *The dictators will exhibit more altruistic behavior in the dictator game under the eye conditions compared to the flower conditions*.

Current research suggests that the psychological mechanisms of the watching eyes effect in prosocial behavior include the “Reputation-Benefit Model” and the “Norm-Punishment Model” (Kawamura and Kusumi, 2017). One possible explanation is that eye cues serve as an implicit social supervision, implying that an individual is being observed and supervised, thereby triggering an individual's reputation management. A positive reputation can elevate an individual's status within a group, making them more likely to be chosen by others as an interaction partner, thereby increasing potential future benefits. Therefore, in the presence of eye cues, individuals may exhibit prosocial behavior in order to maintain a good reputation. Another possible explanation is that eye cues highlight the social norms of the group in a given situation, prompting individuals to behave in accordance with group norms to avoid potential punishment. For instance, in situations where social rules are very clear, such as paying for drinks (Bateson et al., 2006) or waste sorting (Bateson et al., 2013), eye cues make individuals more compliant with social rules. Therefore, eye cues may activate an individual's rule awareness, leading them to behave more in line with established norms (Kawamura and Kusumi, 2017).

The watching eyes effect in prosocial behavior is unstable and can be influenced by factors such as situational anonymity (Tane and Takezawa, 2011), cue presentation duration (Sparks and Barclay, 2013), power motivation (Wang and Dai, 2020), and task type (Baillon et al., 2013). Regarding the manipulation of cue presentation methods in studies on the watching eyes effect in prosocial behavior, the majority of eye cues are presented visually through images: human eye images (Bateson et al., 2006; Ernest-Jones et al., 2011), abstract eye images (Haley and Fessler, 2005; Mifune et al., 2010; Oda et al., 2011), robot resembling human eyes (Burnham and Hare, 2007), and even images featuring facial features composed of three dots (Rigdon et al., 2009; Xin et al., 2016). Other manipulations include the presence of real people in the environment (Lamba and Mace, 2010), or auditory interventions, such as wearing earmuffs to reduce the sound that implies social presence (Haley and Fessler, 2005). The presence of real people nearby encourages individuals to exhibit more prosocial behavior, and the more people present, the more pronounced the watching eyes effect. However, research on the manipulation of auditory social presence cues found no significant difference in altruistic behavior between conditions with and without earmuffs (Haley and Fessler, 2005). Therefore, the presentation of social presence cues through different perceptual channels may have varying effects on the watching eyes effect in prosocial behavior.

Previous studies have manipulated social presence cues using visual images, full-sensory real people, and auditory attenuation to explore the watching eyes effect in prosocial behavior. There has been no use of higher-level verbal cognitive processes to manipulate social presence cues to investigate the watching eyes effect in prosocial behavior. By validating the manipulation of eye cues through imagination, we aim to explore its impact on altruistic behavior. Imagination is the process by which the human brain processes and transforms existing representations to create new images, characterized by its vividness, novelty, and creativity. Although imagination is a top-down cognitive processing method, it has a cognitive model pathway highly similar to visual representation (Tong, 2013) and brain processing areas that are highly correlated (Breedlove et al., 2020). Therefore, exploring the watching eyes effect of altruistic behavior through imagination, on the one hand, further verifies the robustness of the watching eyes effect; on the other hand, it helps further reveal the cognitive mechanisms by which the watching eyes effect operates. Research on the effectiveness of imagined eyes for behavioral intervention will further reduce the intervention costs of altruistic behavior in social behavior, providing more solutions for community management and social governance.

Imagined and visual image stimuli share a high degree of similarity in neural networks. Moreover, imagined eyes might be more vivid and lively than those from visual images, more effectively inducing individuals to anticipate potential future scenarios and fear possible punishments. Previous research has shown that manipulations involving imagination lead to increased self-control in individuals (Yi et al., 2016), a decrease in social discounting (Yi et al., 2016), and an enhancement of prosocial intentions (Gaesser and Schacter, 2014). For instance, Gaesser and Schacter (2014) found that having participants imagine helping others or recalling events related to helping others strengthened their prosocial intentions to assist others. Script theory (Tomkins, 1978) posits that imagined eyes enables individuals to form scripts related to events, and these pre-established scripts directly guide individuals' behavioral performances in subsequent real-life situations. The formation of scripts emphasizes potential future reputations and social normative standards, leading to more prosocial behavior. Therefore, we propose research Hypothesis 1b: *Imagination will lead the dictators in the dictator game to exhibit more altruistic behavior than visual*.

Prosocial behavior is often measured using various game paradigms to study the relationships and dynamic changes between individuals in different situations and within groups. The dictator game is one of the commonly used paradigms to measure individual generosity (Thielmann et al., 2021). In the structure of the dictator game, the dictator holds absolute dominance in the situation, while the recipient can only passively accept. Previous research has focused on the behavior of the dictator in the dictator game, without exploring the impact of different social presence cues on the psychology or potential subsequent behavior of the recipient. However, the recipients' satisfaction with the opponent and the allocation outcome directly affects the future interactions and stability of the relationship between the dictator and the recipient (Van Dijk and De Dreu, 2021). Another innovative aspect of this study is the exploration of the impact of social presence cues on psychological variables of the recipient, such as satisfaction with the opponent's behavior and satisfaction with the allocation outcome. As we suggested in Hypothesis 1a, we assume that the watching eyes effect will be observed in our study. That is, under the eye condition, the dictators will allocate more to the recipients compared to in the flower condition. Higher payoff brings higher satisfactions in the interaction (Frijters et al., 2004). Thus, we propose our research Hypothesis 2a: *under the eye condition, compared to the non-eye condition, the recipients' satisfaction with the dictators' behavior and the allocation outcome will be higher*. In the same vein, we propose our research Hypothesis 2b: *In the imagined conditions, the recipients will report a higher satisfactions with the dictators' behavior and the allocation outcome than in the visual conditions*.

Previous research predominantly utilized the dictator game paradigm without communication (Haley and Fessler, 2005). On one hand, the introduction of communication poses high requirements for experimental platforms and design, making it challenging to implement. On the other hand, analyzing the content of communication is a qualitative analysis, and extracting content from quantitative research is also a challenge. Another innovative aspect of this study is the exploration of differences in communication between the dictator and the recipient in the dictator game under various cue types and presentation mode conditions. Communication can promote prosocial behavior, especially task-related communication, as it can evoke trust-based social norms, leading individuals to behave more cooperatively (Balliet, 2010; Cohen et al., 2010). No prior research has explored whether social presence cues would influence the communication between the dictator and the recipient in the dictator game. Analyzing the differences in communication under various conditions can provide a better understanding of the cognitive processes triggered by eye cues during game interactions. In game paradigms, common communication can be broadly categorized into three types: words that evoke politeness norms, emotional words that express feelings, and words related to task allocation norms. Given that the core measure of this study's dictator game paradigm is altruistic behavior, words related to task allocation norms are further divided into altruistic norm words and egoistic norm words. The “altruistic norm words” are defined as expressions that convey benefits to others, such as “split equally” or “half for each”. In contrast, “egoistic norm words” refer to phrases that imply self-benefit, such as “all for me” or “split 70–30”. Under the eye condition, compared to the non-eye condition, there would be a higher usage of task-related norm phrases. This study preliminarily explores the impact of social presence cues on the communication between the dictators and the recipients. Based on the above, we propose Hypothesis 3a: *Compared to the flower conditions, there will be a higher frequency of expressing altruistic norm words and a lower frequency of egoistic norm words in the eye conditions*. Moreover, another aim of our study is to examine the effect of presentation mode on communication between the dictator and the recipient. Therefore, we propose Hypothesis 3b: *Compared to the visual conditions, there will be a higher frequency of expressing altruistic norm words and a lower frequency of egoistic norm words in the imagined conditions*.

## 2 Method

### 2.1 Participants

We adopted G*Power 3.1 (Faul et al., 2007) to infer required sample size to reach enough statistic power. We set a median effect size (*f* = 0.25), and a statistical significance level α = 0.05, with a two-factor between-subject experimental design. After calculation, we inferred that we required 128 dictators (256 participants in total) to achieve a power of 1−β = 0.80. We recruited 302 undergraduate students, none of whom were majoring in psychology, from a university to participate in an offline laboratory experiment. (*M*_age_ = 19.41, *SD*_age_ = 1.63, *n*_female_ = 183, *n*_male_ = 119). Two pairs of those participants were missing for the dictator game data. Thus, their data were not entered for further data analysis. Our final data included 298 participants (*M*_age_ = 19.41, *SD*_age_ = 1.63, *n*_female_ = 180, *n*_male_ = 118). Participants were randomly assigned into the Imagined Flower (*n* = 74), the Imagined Eye (*n* = 80), the Visual Flower (*n* = 72), and the Visual Eye (*n* = 72) conditions. In each condition, half were dictators and half were recipients. This study were obtained approval of research committee. All participants have signed consent inform and read study information notice. Participants were paid on the basis of their tokens received in dictator game at the exchange rate of 10:1, that is, 10 tokens equals to 1 yuan.

### 2.2 Design

This study employed a 2 (Presentation Mode: Imagined/Visual) × 2 (Cue Type: Eye/Flower) between-subject experimental design. The dependent variables included the altruistic behavior of the dictator, psychological measures of the recipient, and the communication between the dictator and the recipient. The operational definition of altruistic behavior is the amount of tokens that the dictator allocated to the recipient.

The psychological measures for the recipient involved satisfaction with the opponent's behavior and satisfaction with the allocation outcome. Those measures were rated on a Likert 5-point scale. The specific questions were as follows: “*Your satisfaction with the opponent's behavior”*, and “*Your satisfaction with the outcome.”* For each question, five response options were available: 1 *Very Dissatisfied*, 2 *Somewhat Dissatisfied*, 3 *Neutral*, 4 *Somewhat Satisfied*, and 5 *Very Satisfied*.

### 2.3 Materials

#### 2.3.1 Cue types

**Figure 1:**
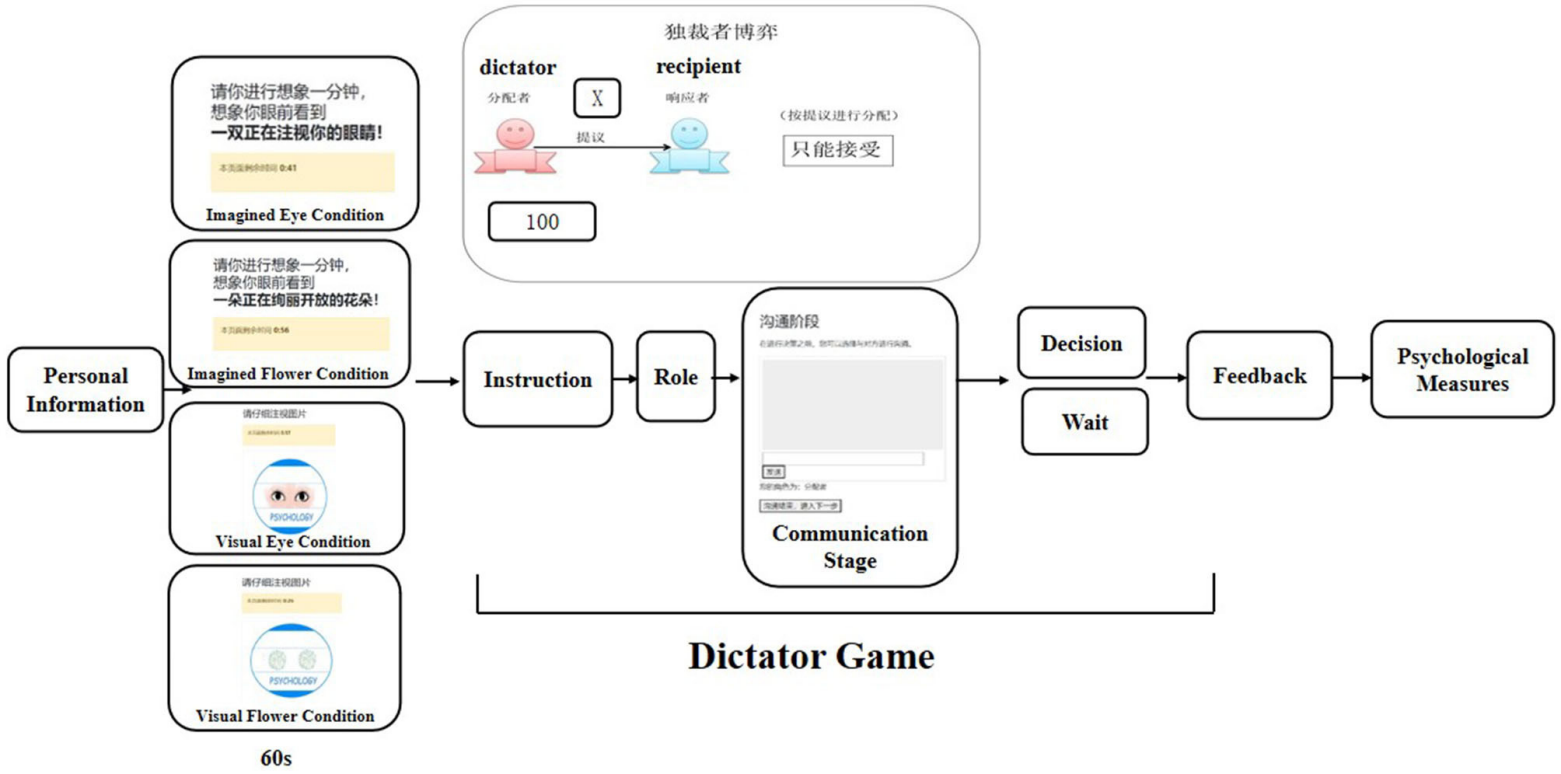
Experimental procedure.

As illustrated in Figure 1, we adopted Wang and Dai (2020) materials (eye and flower pictures) as our stimulus. The imagined eyes is “*Please imagine for a minute, imagine* ***a pair*** ***of eyes in front of you staring at you*.**” The imagined flower is “*Please imagine for a minute, imagine* ***seeing a flower blooming brilliantly***.”

#### 2.3.2 Dictator games with communication stage

The dictator game in our experiment is a one-shot game. The task involves two players: one acting as the dictator and the other as the recipient. The dictator decides how many out of 100 tokens to keep for themselves. The recipient does not have the right to reject the dictator's decision. Before making their decisions, the program enables both the dictator and the recipient to communicate with each other by entering text in an online communication dialogue.

### 2.4 Procedure

**Figure 2:**
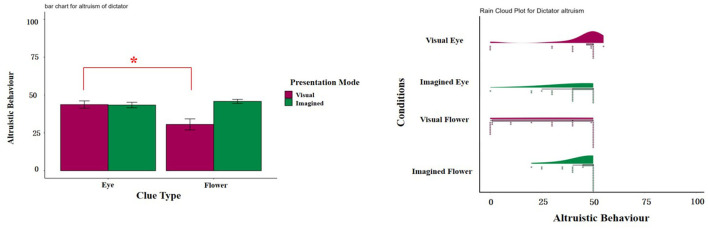
**(Left)** Box plot of the amount allocated by the dictator to the recipient under various conditions. **(Right)** Rain-cloud plot depicting the amounts allocated by the dictator to the recipient under various conditions. The error bars denote 1 Standard Error (SE). ***p < 0.001, **p < 0.01, *p < 0.05.

The experimental procedure is illustrated in Figure 2. We recruited twelve participants per session to partake in the experiments simultaneously. Upon entering the laboratory, each participant received a note indicating their virtual room number and label, such as “Room 1, s001”, where “1” represents the virtual room number and “s001” the label number. Virtual rooms were created by the program, accommodating six participants each. Both the room number and label were assigned randomly, and participants were only aware of their own room and label. To log in and join the experiment, participant assessed a website (e.g., http://152.136.205.120/room/room1). Each virtual room had a unique link; for instance, Room 2's link was http://152.136.205.120/room/room2.

Upon logging in, participants entered their label number to initiate the experiment. In each virtual room, two participants were paired–one was dictator and the other was recipient. They were assigned to one of the experimental conditions: Imagined Flower, Imagined Eye, Visualized Flower, or Visualized Eye.

Participants read study information notice, and signed the informed consent. In response to concerns about demand effects, we ensured that all instructions provided during the experiment were strictly neutral, focusing solely on decision-making tasks rather than cooperative tasks. Despite the possibility of participants attempting to infer the experiment's purpose, the design's between-subject nature precluded them from accessing information about other groups, thereby reducing potential bias in their behavior and responses. Then, the participants provided some personal information, such as participants number, age, and gender. After both paired participants had completed their personal information, they were exposed to different cue types for 60 s. In visual conditions, participants were presented with either a pair of eyes or a flower picture, while in imagined conditions, they received imagination induction descriptions.

Next, they engaged in the dictator game, roles assigned by the program. Participants received instructions for the dictator game, and became aware of their role. They proceed to an intermediary page for 5 s. This page displays the message “Please ready yourself for entering the stage where you will communicate with other participants.” This leads to the communication stage. During the communication stage, dictators and recipients were allowed to exchange text messages. The dictator then decided how many of the 100 tokens to keep for themselves, while the recipient awaited the dictator's decision.

Subsequently, participants entered into results feedback stage. Next, they answered questions about their satisfactions with the opponents, and the allocation outcome. Finally, they were compensated based on their choices in the dictator game.

## 3 Results

### 3.1 Effect of presentation mode and cue type on dictators' altruistic behavior

Table 1 presented the mean and standard deviation of dictator's allocation across four different experimental conditions. To test effect of presentation mode and cue type on dictators' altruistic behavior, we conducted a two-way ANOVA on the amount of tokens that dictator assigned to recipient. We found that the main effect of presentation mode was significant, $F_{\left(1,145\right)}=9.49,p=0.002,\eta_{p}^{2}=0.06$. The altruistic behaviors in dictator in the imagined conditions (*M* = 44.55, *SD* = 9.97) were significantly higher than that in the visual conditions (*M* = 37.14, *SD* = 19.58), *p* = 0.003. The main effect of cue type was significant, $F_{\left(1,145\right)}=4.87,p=0.029,\eta_{p}^{2}=0.03$. The altruistic behaviors in dictator in the eye conditions (*M* = 43.53, *SD* = 12.84) were significantly higher than that in the flower conditions (*M* = 38.30, *SD* = 18.04), *p* = 0.032.

**Table 1:** Mean and standard deviation of dictator's allocation across four conditions.

**Conditions**	***n***	***M***	***SD***	**95%CI**
Imagined eye	40	43.38	11.39	[39.73, 47.02]
Visual eye	36	43.69	14.44	[38.81, 48.58]
Imagined flower	37	45.81	8.12	[43.10, 48.52]
Visual flower	36	30.58	21.93	[23.16, 38.00]

*M* and *SD* represents mean and standard deviation, respectively.

The interaction between presentation mode and cue type were significant, $F_{\left(1,145\right)}=10.32,p=0.0016,\eta_{p}^{2}=0.07$, as shown in Figure 2. *Post-hoc* test found that under the visual conditions, dictators' altruistic behavior between the eye (*M* = 43.39, *SD* = 14.44, 95%CI [38.81, 48.58]) were significantly higher than the flower conditions (*M* = 30.58, *SD* = 21.93, 95%CI [23.16, 38.00]). However, under the imagined conditions, there were no significant difference on dictators' altruistic behavior between the eye and flower conditions, *p* = 0.47.

### 3.2 Effects of presentation mode and cue type on recipients' psychological variables

A two-way ANOVA was conducted with the presentation mode and cue type as independent variables, focusing on the psychological variables subjectively rated by the recipients. The psychological variables are satisfaction with the dictators' behavior, and satisfaction with the allocation outcome. Table 2 showed the mean and standard deviation of recipients' psychological variables across four conditions.

**Table 2:** Mean and standard deviation of recipients' psychological measures across four conditions.

		**Satisfaction with the dictator's behavior**		**Satisfaction with allocation outcome**		**Payoff**	
**Conditions**	***n***	***M***	***SD***	***M***	***SD***	***M***	***SD***
Imagined eye	40	4.1	1.15	4.1	1.06	43.38	11.39
Visual eye	36	4.22	1.27	4.28	1.16	43.69	14.44
Imagined flower	37	3.86	1.46	3.86	1.49	45.81	8.12
Visual flower	36	3.61	1.63	3.5	1.63	30.58	21.93

#### 3.2.1 Recipients' satisfaction with the dictator's behavior

The results indicated that when assessing satisfaction with the dictators' behavior as the dependent variable, the main effect of cue type was not significant, $F_{\left(1,145\right)}=3.49,p=0.064,\eta_{p}^{2}=0.02,95\%\text{CI}\;\left[0.00,0.08\right]$. The main effect of the presentation mode was not significant, $F_{\left(1,145\right)}=0.08,p=0.772,\eta_{p}^{2}<0.001$. The interaction between presentation mode and cue type was not significant, $F_{\left(1,145\right)}=0.69,p=0.408,\eta_{p}^{2}<0.001$.

#### 3.2.2 Recipients' satisfaction with allocation outcome

For the dependent variable satisfaction with the allocation outcome, results showed: The main effect of cue type was significant, $F_{\left(1,145\right)}=5.24,p=0.024,\eta_{p}^{2}=0.03,95\%\text{CI}\;\left[0.00,0.10\right]$. Under the eye condition (*M* = 4.18, *SD* = 1.10), recipients were more satisfied with the allocation outcome than under the flower condition (*M* = 3.68, *SD* = 1.56), *p* = 0.0255. The main effect of the presentation mode was not significant, $F_{\left(1,145\right)}=0.18,p=0.673,\eta_{p}^{2}<0.001$. The interaction between presentation mode and cue type was not significant, $F_{\left(1,145\right)}=1.50,p=0.222,\eta_{p}^{2}=0.01$.

### 3.3 Effect of presentation mode and cue type on the communication between dictator and recipient

To explore whether there are differences in the communication between dictators and recipients in the dictator game under various presentation modes and cue types, we conducted a text analysis of the communication under different conditions. Using the *jiebaR* package in R (Qin and Wu, 2019), we first segmented the Chinese communication text based on different conditions, and then counted the word frequency (see Table 3). Following the methodology of Cohen et al. (2010), we classified the communication text into four categories: daily politeness words, emotional expression words, altruistic norm words, and egoistic norm words. We then counted the word frequency for these categories and conducted Kruska-Wallis chi-square test compare the four types of words under four conditions.

**Table 3:** Frequency of different categories of communication between dictators and recipients.

**Conditions**	**Daily politeness**	**Emotional expression**	**Altruistic norm**	**Egoistic norm**
Visual eye	37	27	30	14
Imagined eye	32	47	27	13
Visual flower	39	28	35	32
Imagined flower	33	14	24	7

Daily politeness words include greetings (such as “hello”, “hi”, etc.), appellative terms (such as “you”, “brother”, etc.), and other polite expressions (such as “thank you”, “may I ask”, etc.). Emotional expression words encompass non-meaningful spoken emotions, that may not convey the speaker's actual emotions clearly and lack a specific, directed meaning in context, daily emotional words (such as “hahaha”, “laugh to death”, “emoticons”, etc.), and adjectives with clear emotional connotations (such as “like”, “cruel”, etc.). It is important to note that this experiment did not distinguish the value of emotional expression words; the word frequency represents the extent to which participants are willing to reveal their emotions in communication. Altruistic norm words refer to expressions that benefit others (such as “split equally”, “half for each”, etc.), while egoistic norm words include phrases that benefit oneself (such as “all for me”, “split 70–30”, etc.).

The frequency of different categories of words under different conditions is presented in Table 3. We have conducted several Kruska-Wallis test for the frequency of different categories. The results revealed that the main effect of Presentation Mode on the frequency was not significant, $\chi_{\left(1\right)}^{2}=0,p=1$; the main effect of Cue Type on the frequency was not significant, $\chi_{\left(1\right)}^{2}=3.201,p=0.0735$; the main effect of Category was not significant, $\chi_{\left(3\right)}^{2}=6.597,p=0.085$.

#### 3.3.1 Daily politeness words

A χ^2^-test was conducted for the four conditions. The results revealed no significant difference in the frequency of daily politeness words in the communication between dictators and recipients across the four conditions, $\chi_{\left(3\right)}^{2}=0.647,p=0.886$.

#### 3.3.2 Emotional expression words

For the four conditions, the frequency of emotional expressions words in the communication texts of the Imagined Flower condition was significantly lower than the other three conditions [$\chi_{\left(3\right)}^{2}=19.103,p<0.001$]. The frequency of emotional expression words in the communication texts of the Imagined Eye condition was significantly higher than the other conditions [$\chi_{\left(2\right)}^{2}=7.471,p=0.024$]. There was no significant difference between the Visualized Flower condition and the Visualized Eye condition [$\chi_{\left(1\right)}^{2}=0.018,p=0.893$].

#### 3.3.3 Altruistic norm words

Across the four conditions, there was no significant difference in the frequency of altruistic norm words in the communication texts, $\chi_{\left(3\right)}^{2}=2.276,p=0.517$.

#### 3.3.4 Egoistic norm words

For the four conditions, the frequency of egoistic norm words in the communication texts of the Visualized Flower condition was significantly higher than the Visualized Eye condition, the Imagined Eye condition, and the Imagined Flower condition [$\chi_{\left(3\right)}^{2}=21.152,p<0.001$].

## 4 Discussion

This study found that: (1) The interaction between presentation mode and cue type significantly affected altruistic behavior of the dictator in the dictator game. Under the visual condition, the eye group exhibited a higher level of altruistic behavior than the flower group. (2) When the dependent variable was the recipient's satisfaction with the other's behavior, none was significant. When the dependent variable was satisfaction with the allocation outcome, only the main effect of cue type was significant. Specifically, under the eye condition, satisfaction with the allocation outcome were higher than under the flower condition. (3) Moreover, the results shows that when the dependent variable was egoistic norm words, under the visual flower condition, compared to the other three conditions, the communication between dictators and recipients used more egoistic norm words.

### 4.1 Dictators' altruistic behavior

The results supported Hypothesis 1a. Presenting eye cues visually can promote altruistic behavior, consistent with previous research (Haley and Fessler, 2005; Oda et al., 2011). The results supported Hypothesis 1b. Under imagined conditions, dictators exhibited more altruistic behavior than visual conditions. To some extent, this verified the robustness of the watching eyes effect under the manipulation of higher-level verbal cognitive processes. However, when eye cues are presented through imagination, there was no significant difference in altruistic behavior between the imagined eyes group and the imagined flowers group. A plausible explanation is the interaction between the concept of the flower and the act of imagination, which might lead to an increase in altruistic behavior in the imagined flowers group. Additionally, the presence of imagined eye cues could have amplified altruistic tendencies. Considering that the control group involved imagining flowers, it is possible that participants associated this with positive personal experiences, as suggested by Weinstein et al. (2009) and Guéguen (2012). Such positive personal experience could trigger positive emotions and, consequently, enhance altruistic behavior, aligning with findings by Aknin et al. (2018) and Mesurado et al. (2021). Thus, this interaction might account for the heightened altruism observed in both conditions.

Alternatively, imagination itself can increase altruistic behavior, regardless of the content of the imagination (eyes or flowers). The method of imagination, regardless of the content required for the participants to imagine, provides room for imagination and can promote individuals to be more altruistic. This result is consistent with the positive effects of mindfulness and meditation on individual self-affirmation (Cohen and Sherman, 2014). When individuals engage in imaginative activities, they gain more powerful energy, which allows them to exhibit more other-oriented behavior when dealing with the external world or socializing with others (Crisp and Turner, 2012).

### 4.2 Recipients' psychological variables

The research findings partially support Hypothesis 2a and do not support Hypothesis 2b. Specifically, under the eye conditions, the recipients' satisfaction with the allocation result was found to be higher compared to the flower conditions. A possible reason is that under the eye condition, the recipients' gains are greater than under the flower condition. Therefore, this is consistent with expectations. The greater the gain, the more satisfied the recipient is with the allocation result. However, compared with visual conditions, recipients were not more satisfied with the dictators' allocation outcome under imagined conditions even though they received more tokens. A plausible reason is that the satisfaction with allocation outcome is multifaceted, with procedural fairness being a significant factor (Van den Bos et al., 1998). Procedural fairness concerns the transparency and equity of the distribution process (Garćıa-Izquierdo et al., 2012; Bakotić and Bulog, 2021). Since our recipients were informed about the allotment process and had communicated with the dictator, their satisfaction with the outcome likely hinged on their perception of procedural fairness, rather than the quantity of tokens received.

Moreover, our findings revealed no significant main effects of cue type and presentation mode on recipients' satisfaction. Nevertheless, it is noteworthy that 52.3% of recipients (*n* = 78) reported “*5 - very satisfied*” regarding the dictators' behavior, indicating a high level of satisfaction. This could be attributed to the collectivist culture of China, which values social harmony (Markus and Kitayama, 1991), and a tendency for individuals to exhibit friendly behaviors (Rego and Cunha, 2009). Furthermore, the homogeneity of the sample, comprising students from the same educational institution, may lead to more favorable evaluations among in-group members, as suggested by Brewer (1999).

### 4.3 Communication between the dictator and the recipient

Whether the activation of different types of social presence cues will affect the communication process in the dictator game is still unknown, and no research has explored it. The results of this study found that there is no significant difference in the use of polite phrases under the four conditions. In terms of emotion-related phrases, there are significant differences among the four conditions. Specifically, compared to the other three conditions, the communication text in the imagined eyes group uses more emotional words, while the imagined flowers group uses fewer emotional phrases. In terms of task-related phrases, we further divided them into altruistic norm words and egoistic norm words. The results showed that there was no significant difference between the four different conditions in terms of altruistic norm words, but in terms of egoistic norm words, the frequency in the visual flower group was significantly higher than in the other three conditions. This finding is partially consistent with Hypothesis 3a. Only in the communication text of the imagined eyes group and the imagined flowers group, the eye group used more emotion-related phrases than the flower group. But there is no significant difference between the visual eye group and the visual flower group. Furthermore, we hypothesized that the eye group would express more altruistic norm words and fewer self-serving norm words than the flower group. Our data results found that there was no significant difference in altruistic norm words, but in terms of egoistic norm words, the frequency in the visual flower group was the highest.

### 4.4 Limitations and prospects

This study has certain limitations. Firstly, our results can find the impact of different cue presentation modes and cue types on the communication text of dictators and recipients in the dictator game. While we observed the effects of different presentation modes and cue types on the altruistic behavior and psychological variables of both dictators and recipients in the dictator game, we could not establish a causal relationship between these factors and the communication text. Consequently, there was no statistical analysis correlating the frequency of emotion words with the dictators' allocations, given the absence of significant differences between the imagined eye and imagined flower groups. We recommend that future research endeavors to delineate the causal links between communication text in the dictator game with communication and individual behavior. Previous research, such as the study by Yamamori et al. (2008), has shown that if recipients can make non-task-related requests to dictators before the dictators' decision, dictators tends to provide more benefits to recipients. Exploring this further could effectively determine whether social presence cues continue to influence behavior in the dictator game with communication.

Secondly, the altruistic behavior examined in this study adheres to the traditional dictator game paradigm. Notably, some studies, such as Dreber et al. (2013), have suggested that the social framework effect may not affect behavior in the dictator game, it could affect behavior in the ultimatum game. Future research could integrate the token allocation scenario with more real-life scenarios to enrich the problem's background. This approach would still align with the benefit framework of the dictator game. However, employing different situational narratives could be instrumental in examining how various contextual frameworks impact the influence of imagined eyes on altruistic behavior.

Thirdly, another aspect worth exploring is the impact of the ambiguity of positive evaluations from others on altruistic behavior, as highlighted by Kawamura and Kusumi (2018). Altruistic behavior that remains unseen, and thus unevaluated by others, does not garner positive evaluations. This raises an interesting premise: some altruistic behaviors are perhaps only performed with the knowledge that they will be positively evaluated by others. Conversely, when altruistic behaviors might elicit ambiguous evaluations, there may be a reluctance to publicize these behaviors. Future research should investigate whether the influence of the eye cue varies in situations where the same altruistic behavior could elicit positive evaluations in some contexts and negative evaluations in others.

## 5 Conclusion

The “watching eyes effect” underscores the profound influence of both visual and imagined social cues on human behavior, particularly in the realm of altruism and prosocial actions. This study has illuminated the nuanced ways in which such cues, even when merely imagined, can shape behavior within the context of the dictator game. The pronounced effects observed visual social cues, contrasted with the more subtle impacts in the imagined scenario, highlight the intricate interplay between perception, cognition, and behavior. Furthermore, the distinct communication patterns and satisfaction levels reported by recipients across different conditions emphasize the multifaceted nature of human interactions and the underlying psychological mechanisms underlying them. As society embraces with virtual interactions, understanding such effects becomes paramount. The insights gleaned from this research not only contribute significantly to the academic discourse on prosocial behavior but also offer valuable implications for designing interventions and platforms that foster positive human interactions in both physical and digital environments.

## Data availability statement

The datasets presented in this study can be found in online repositories. The names of the repository/repositories and accession number(s) can be found at: https://osf.io/5xf92/.

## Ethics statement

The studies involving humans were approved by Central University of Finance and Economics, Beijing, China. The studies were conducted in accordance with the local legislation and institutional requirements. The participants provided their written informed consent to participate in this study.

## Author contributions

JL: Conceptualization, Data curation, Project administration, Writing—original draft, Writing—review & editing. YS: Data curation, Visualization, Writing—review & editing. ZH: Writing—review & editing. CZ: Conceptualization, Resources, Writing—review & editing. JM: Resources, Writing—review & editing. HZ: Conceptualization, Funding acquisition, Resources, Supervision, Writing—review & editing.
