# Supplementary material for: Watching eyes effect: the impact of imagined eyes on prosocial behavior and satisfactions in the dictator game
Source: Front Psychol. 2024 Jan 10;14:1292232. doi: 10.3389/fpsyg.2023.1292232 (PMC10806148; doi:10.3389/fpsyg.2023.1292232)
Supplement: Supplementary file 1 [file Data_Sheet_1.PDF]

Experimental Instructions for Imagined Watching Eyes Effect

December 4, 2023

Contents

1 the Visualized Eye condition **2**

2 the Visualized Flower condition **14**

3 the Imagined Eye Condition **26**

4 the Imagined Flower condition **38**

This file provides a detailed description of the impact of the imagined watching eyes effect on behavior and psychological measures in the dictator game. The study encompassed four conditions: the Visualized Eye condition, the Visualized Flower condition, the Imagined Eye condition, and the Imagined Flower condition.

1 the Visualized Eye condition

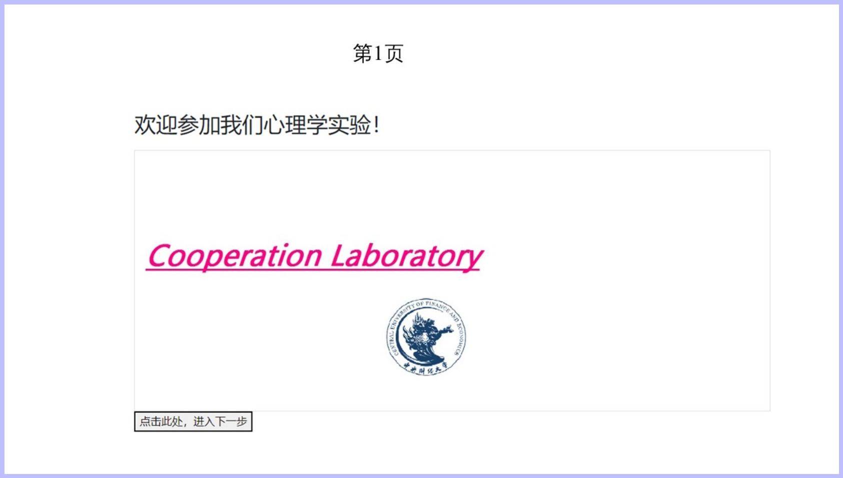

Welcome to our psychology experiments.
Cooperation Laboratory.
Click here to the next page.

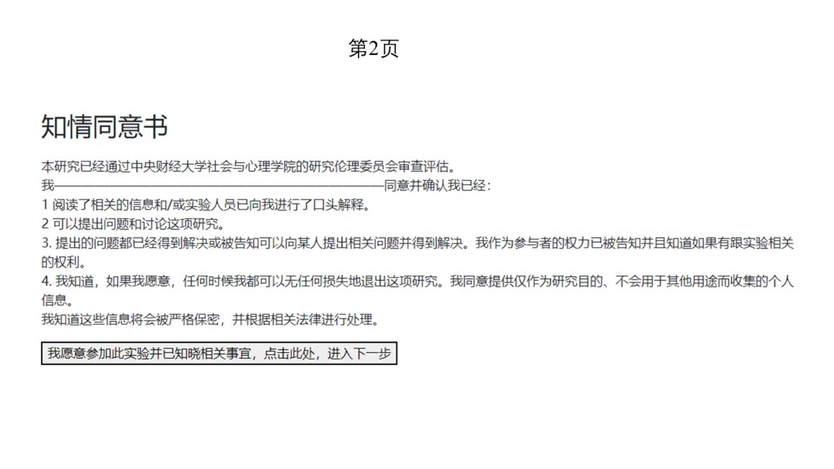
第2页

知情同意书

本研究已经通过中央财经大学社会与心理学院的研究伦理委员会审查评估。
我————————————————————————————同意并确认我已经:
1 阅读了相关的信息和/或实验人员已向我进行了口头解释。
2 可以提出问题和讨论这项研究。
3. 提出的问题都已经得到解决或被告知可以向某人提出相关问题并得到解决。我作为参与者的权力已被告知并且知道如果有跟实验相关的权利。
4. 我知道，如果我愿意，任何时候我都可以无任何损失地退出这项研究。我同意提供仅作为研究目的、不会用于其他用途而收集的个人信息。
我知道这些信息将会被严格保密，并根据相关法律进行处理。

我愿意参加此实验并已知晓相关事宜，点击此处，进入下一步

Informed Consent

This study has been reviewed and approved by the Research Ethics Committee of [anonymous for review reason]. By participating, I confirm that:

1. I have read and understood the relevant information provided about the study, or it has been explained to me verbally by the experimenter.

2. I have the opportunity to ask questions and discuss any aspect of the study.

3. Any questions I have asked have been satisfactorily addressed. I am informed that I can continue to seek clarification and ask further questions as needed. I am also aware of my rights as a participant, including any specific rights related to this experiment.

4. I understand that I am free to withdraw from this study at any time without any penalty. I consent to provide personal information for the purposes of this research, with the assurance that it will be used solely for research purposes and handled with strict confidentiality in compliance with relevant laws.

I agree to participate in this experiment and have been fully informed about its nature. To proceed to the next page, please click here.

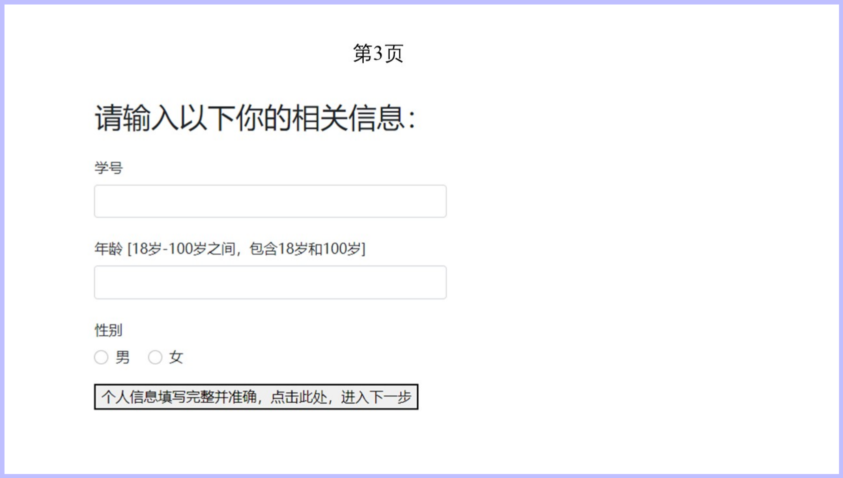

Enter your information:
student ID
Age (18-100 years, including 18 and 100 years old)
Gender: Male; Female.
I have completed my personal information. Click here to proceed to the next page.

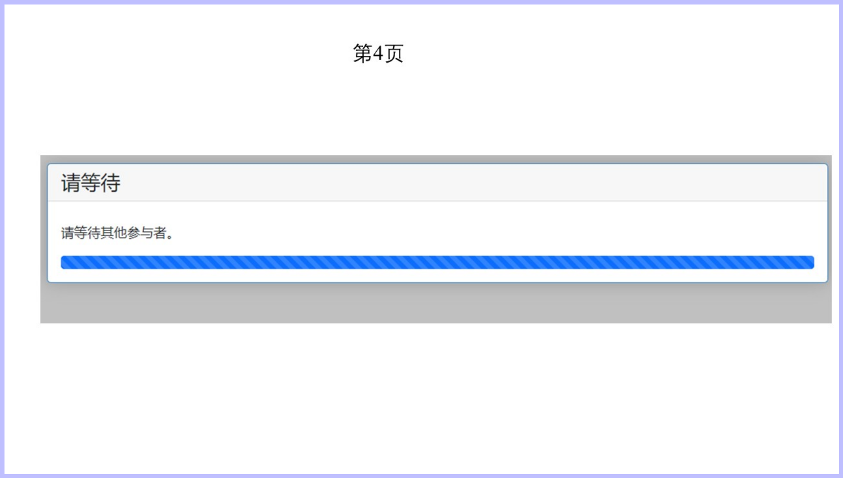

Please wait for other participants.

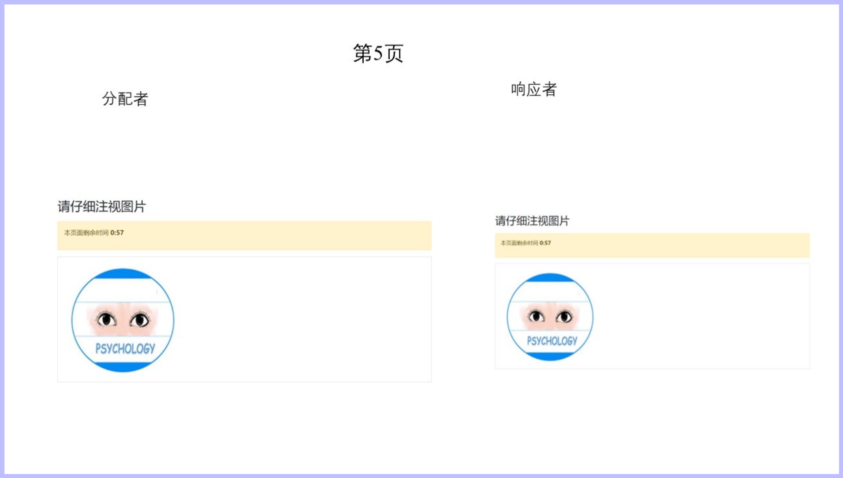

Dictator
Please carefully look at the picture.
Time left for this page: 0:57.

Recipient
Please carefully look at the picture.
Time left for this page: 0:57.

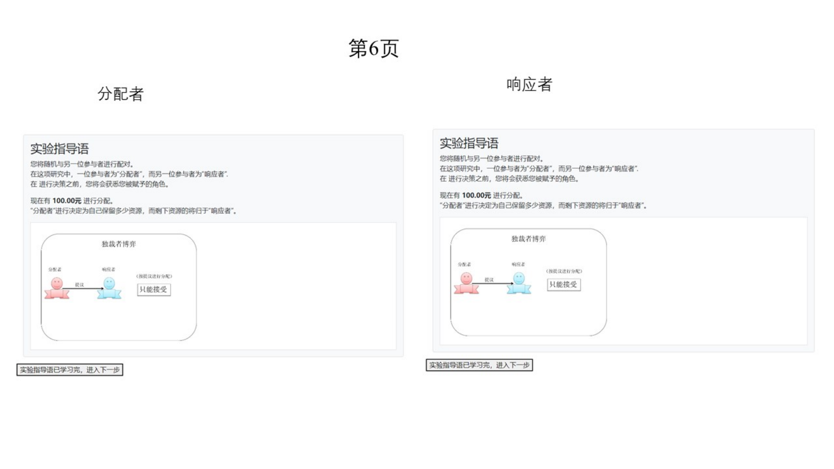

Dictator

Experiment Instructions:
You will be randomly paired with another participant for this study. In this experiment, there will be two roles: one participant will be the "dictator", and the other will be the "recipient".
Before you make any decision, your role in the study will be clearly communicated to you.
A total of **100** RMB will be available for allocation. The dictator will decide the amount of this resource they wish to keep for themselves. The remaining resources will then be allocated to the recipient.
Below, you will find an illustrative picture that briefly describes the dynamics of the Dictator Game: the dictator makes the allocation, and the recipient must accept this decision.
Once you have understood these instructions, please click the button below the picture to proceed to the next page.

Recipient

Experiment Instructions:
You will be randomly paired with another participant for this study. In this experiment, there will be two roles: one participant will be the "dictator", and the other will be the "recipient".
Before you make any decision, your role in the study will be clearly communicated to you.
A total of **100** RMB will be available for allocation. The dictator will decide the amount of this resource they wish to keep for themselves. The remaining resources will then be allocated to the recipient.
Below, you will find an illustrative picture that briefly describes the dynamics of the Dictator Game: the dictator makes the allocation, and the recipient must accept this decision.
Once you have understood these instructions, please click the button below the picture to proceed to the next page.

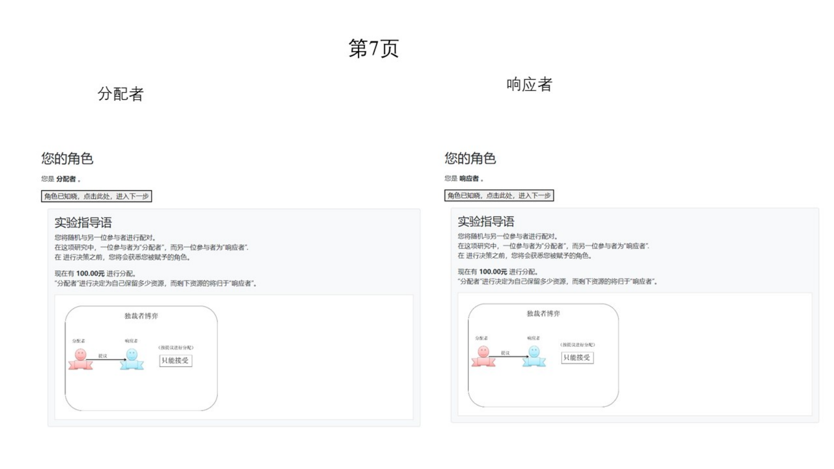

Dictator
Your role is: You are the dictator. Once the role is acknowledged, click here to proceed to the next page.

Recipient
Your role is: You are the recipient. Once the role is acknowledged, click here to proceed to the next page.

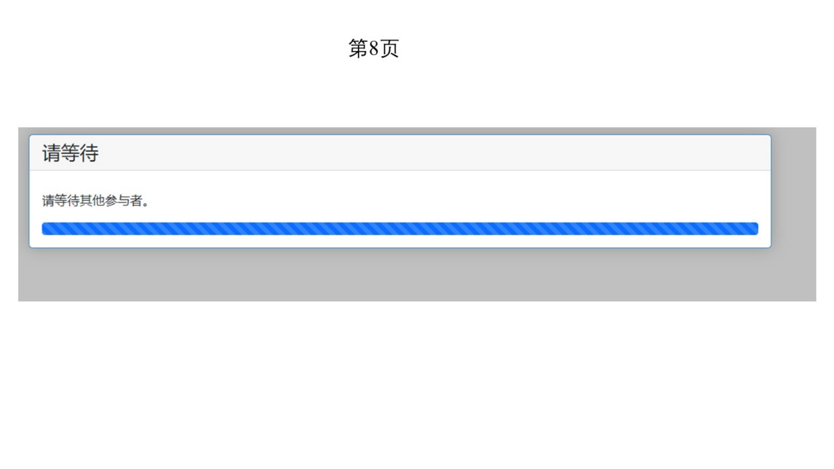

Please wait for other participants.

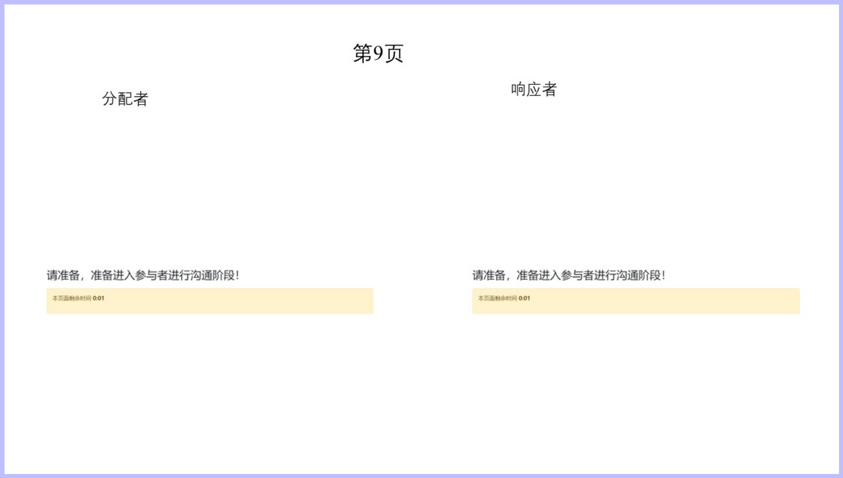

Dictator
Please ready yourself for entering the stage where you will communicate with other participants.
Time left for this page: 0.05

Recipient
Please ready yourself for entering the stage where you will communicate with other participants.
Time left for this page: 0.05

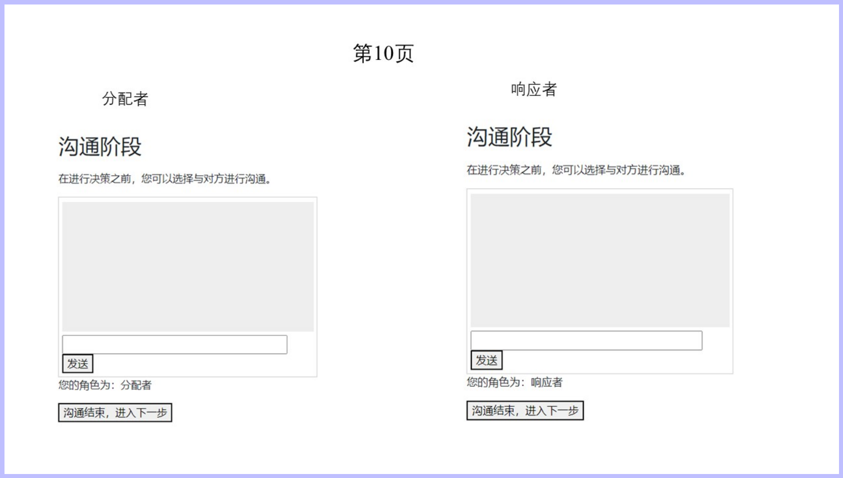

Dictator
Communication Stage:
Before making a decision, you have the option to communicate with the opponent.
Send.
Your role is: **Dictator**.
After communication is finished, proceed to the next step.

Recipient
Communication Stage:
Before making a decision, you have the option to communicate with the opponent.
Send.
Your role is: **Recipient**.
After communication is finished, proceed to the next step.

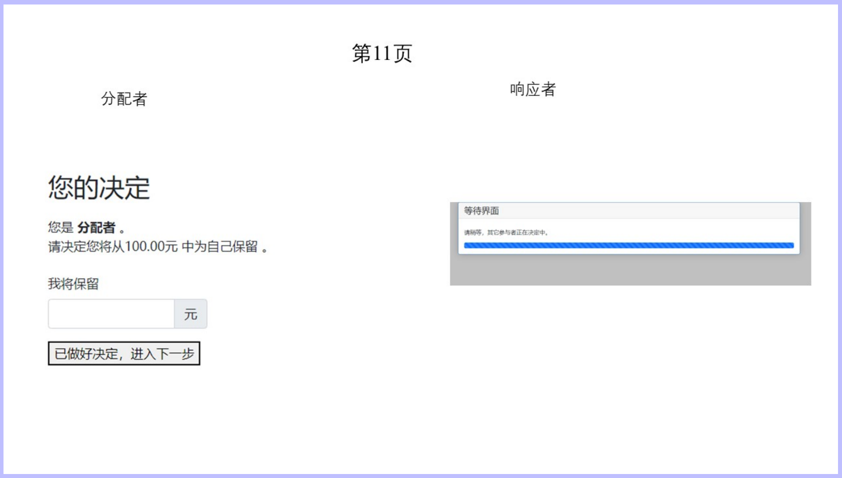

Dictator
Your Decision:
You are the dictator.
You will decide how much of the 100 RMB to keep for yourself.
I will keep:
______ RMB.
The decision has been made; proceed to the next step.

Recipient
Waiting Interface:
Please wait, other participants are making a decision.

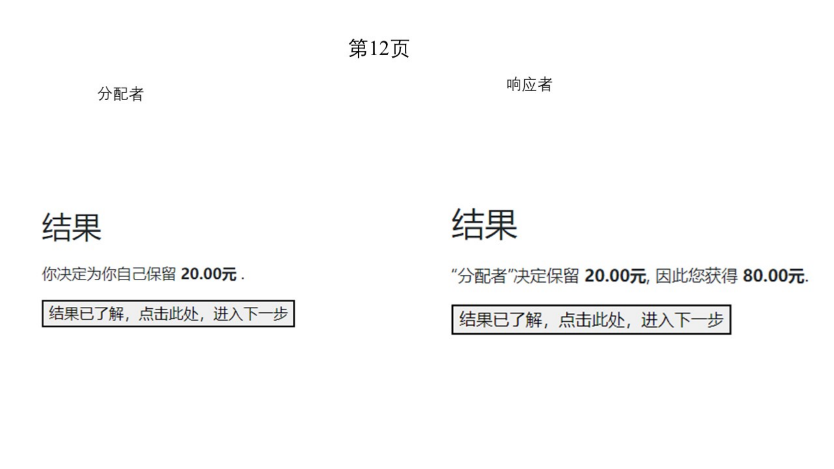

Dictator
Results:
You have decided to keep 20 RMB for yourself.
After you have understood the results, click here to proceed to the next step.

Recipient
Results:
The dictator has decided to keep 20 RMB, so you receive 80 RMB.
After you have understood the results, click here to proceed to the next step.

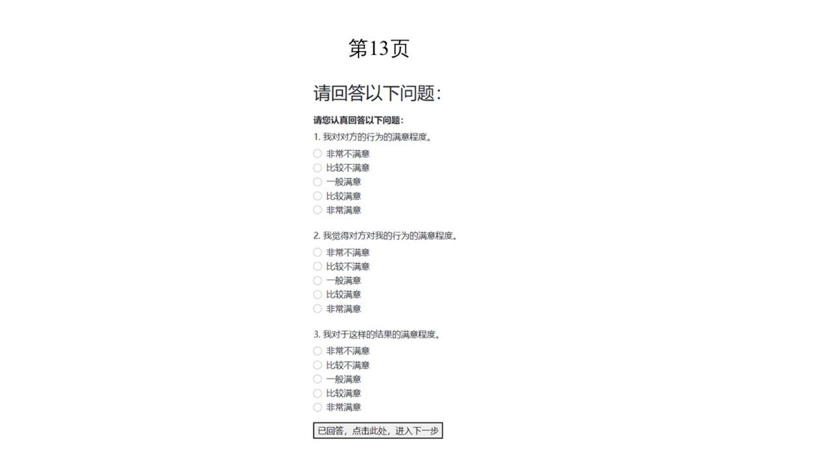
第13页

请回答以下问题:

请您认真回答以下问题:

1. 我对对方的行为的满意程度。

- ○ 非常不满意
- ○ 比较不满意
- ○ 一般满意
- ○ 比较满意
- ○ 非常满意

2. 我觉得对方对我的行为的满意程度。

- ○ 非常不满意
- ○ 比较不满意
- ○ 一般满意
- ○ 比较满意
- ○ 非常满意

3. 我对于这样的结果的满意程度。

- ○ 非常不满意
- ○ 比较不满意
- ○ 一般满意
- ○ 比较满意
- ○ 非常满意

已回答，点击此处，进入下一步

Please thoughtfully answer the following questions concerning:
1. Your satisfaction with the opponent's behavior,
2. Your perception of the opponent's satisfaction with your behavior, and
3. Your satisfaction with the outcome.
For each question, you have five response options: Very Dissatisfied, Somewhat Dissatisfied, Neutral, Somewhat Satisfied, Very Satisfied.
After you have answered these questions, click here to proceed to the next step.

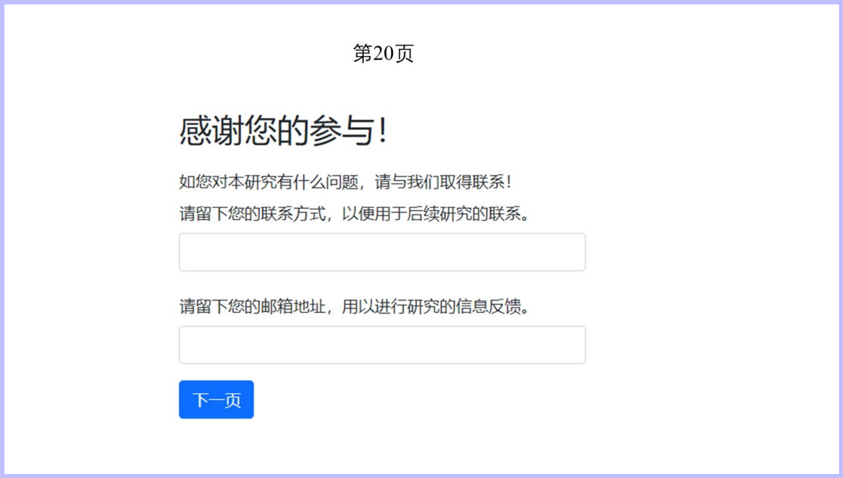

Thank you for your participation.
If you have any questions about this study, please feel free to contact us.
For future research communications, kindly leave your contact number.
Additionally, please provide your email address to receive feedback regarding the study.

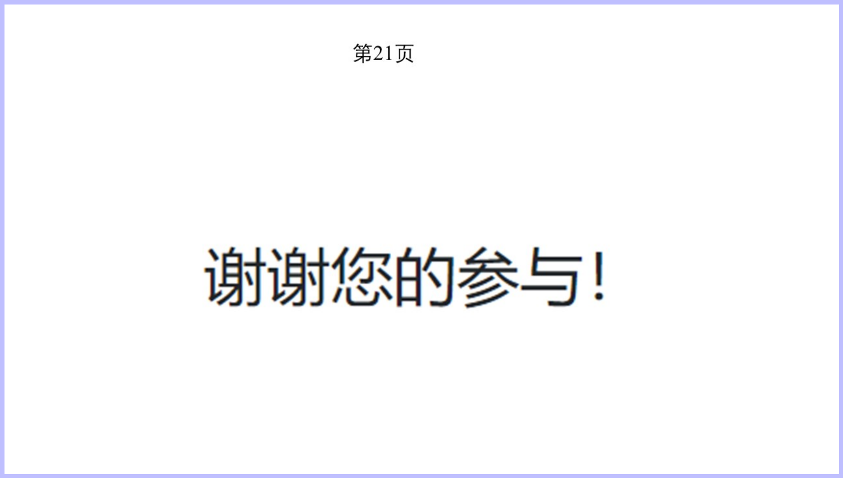

Thank you for your participants.

2 the Visualized Flower condition

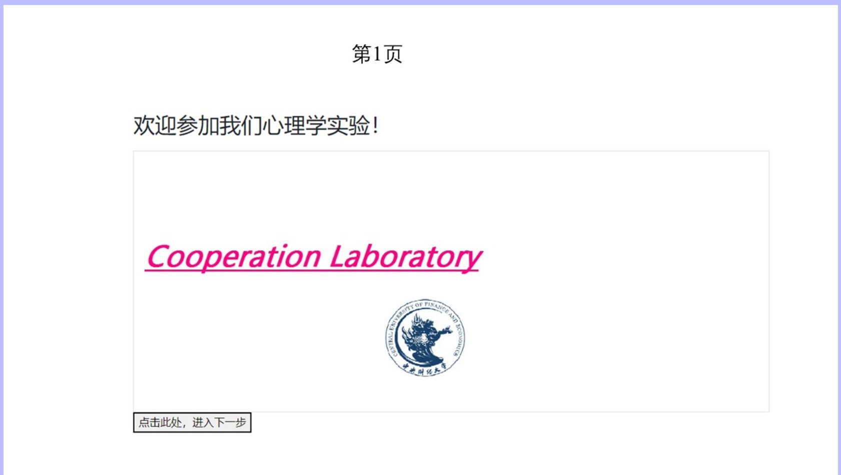

Welcome to our psychology experiments.
Cooperation Laboratory.
Click here to the next page.

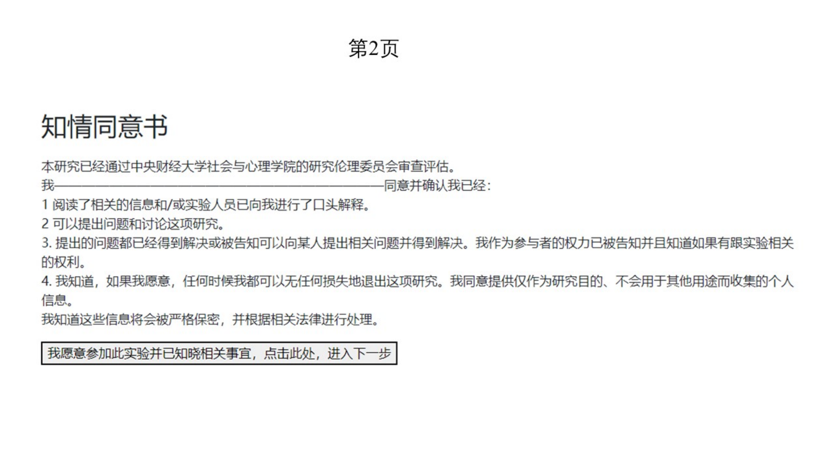

第2页

知情同意书

本研究已经通过中央财经大学社会与心理学院的研究伦理委员会审查评估。
我————————————————————————————同意并确认我已经:
1 阅读了相关的信息和/或实验人员已向我进行了口头解释。
2 可以提出问题和讨论这项研究。
3. 提出的问题都已经得到解决或被告知可以向某人提出相关问题并得到解决。我作为参与者的权力已被告知并且知道如果有跟实验相关的权利。
4. 我知道，如果我愿意，任何时候我都可以无任何损失地退出这项研究。我同意提供仅作为研究目的、不会用于其他用途而收集的个人信息。
我知道这些信息将会被严格保密，并根据相关法律进行处理。

我愿意参加此实验并已知晓相关事宜，点击此处，进入下一步

Informed Consent

This study has been reviewed and approved by the Research Ethics Committee of [anonymous for review reason]. By participating, I confirm that:

1. I have read and understood the relevant information provided about the study, or it has been explained to me verbally by the experimenter.
2. I have the opportunity to ask questions and discuss any aspect of the study.
3. Any questions I have asked have been satisfactorily addressed. I am informed that I can continue to seek clarification and ask further questions as needed. I am also aware of my rights as a participant, including any specific rights related to this experiment.
4. I understand that I am free to withdraw from this study at any time without any penalty. I consent to provide personal information for the purposes of this research, with the assurance that it will be used solely for research purposes and handled with strict confidentiality in compliance with relevant laws.

I agree to participate in this experiment and have been fully informed about its nature. To proceed to the next page, please click here.

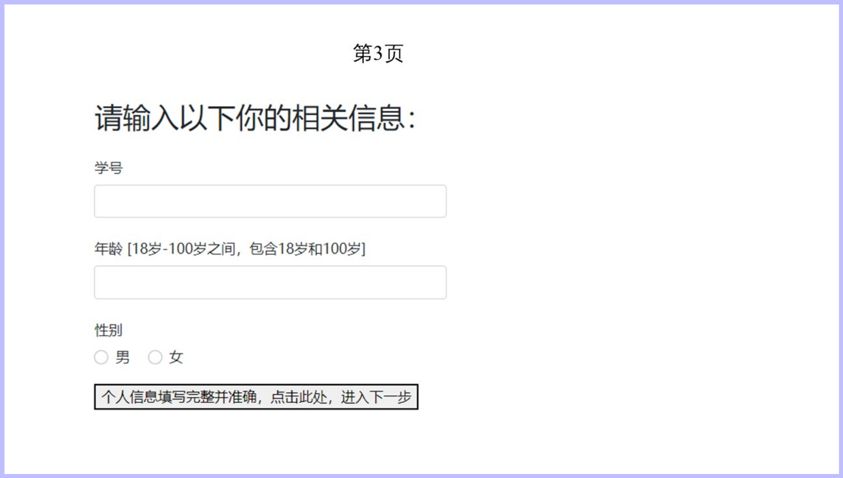

Enter your information:
student ID
Age (18-100 years, including 18 and 100 years old)
Gender: Male; Female.
I have completed my personal information. Click here to proceed to the next page.

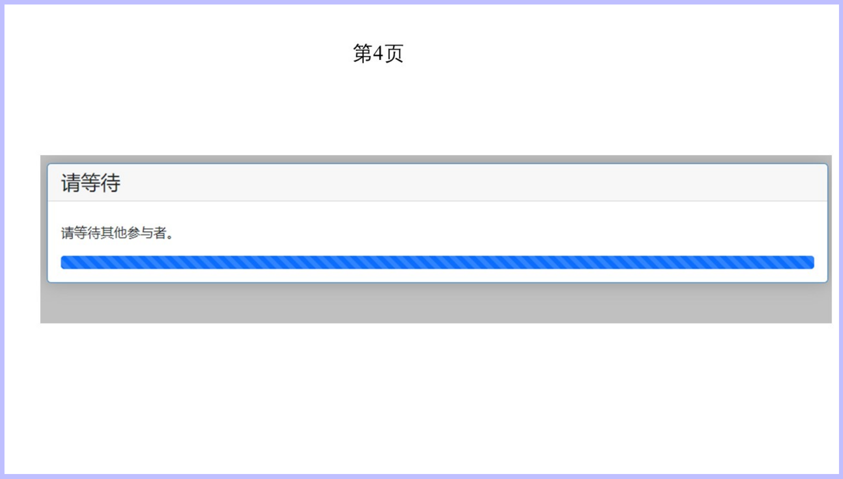

Please wait for other participants.

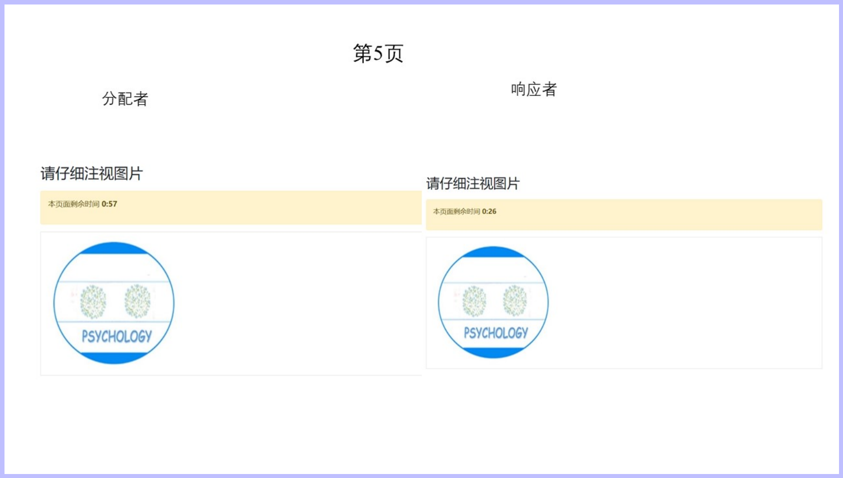

Dictator
Please carefully look at the picture.
Time left for this page: 0:57.

Recipient
The content is the same as the left picture.

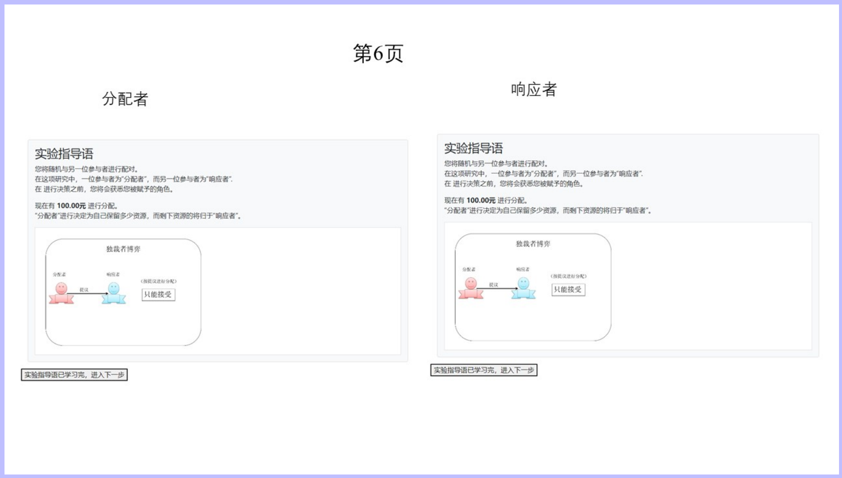

Dictator

Experiment Instructions:

You will be randomly paired with another participant for this study. In this experiment, there will be two roles: one participant will be the "dictator", and the other will be the "recipient".

Before you make any decision, your role in the study will be clearly communicated to you.

A total of **100** RMB will be available for allocation. The dictator will decide the amount of this resource they wish to keep for themselves. The remaining resources will then be allocated to the recipient.

Below, you will find an illustrative picture that briefly describes the dynamics of the Dictator Game: the dictator makes the allocation, and the recipient must accept this decision.

Once you have understood these instructions, please click the button below the picture to proceed to the next page.

Recipient

Experiment Instructions:

You will be randomly paired with another participant for this study. In this experiment, there will be two roles: one participant will be the "dictator", and the other will be the "recipient".

Before you make any decision, your role in the study will be clearly communicated to you.

A total of **100** RMB will be available for allocation. The dictator will decide the amount of this resource they wish to keep for themselves. The remaining resources will then be allocated to the recipient.

Below, you will find an illustrative picture that briefly describes the dynamics of the Dictator Game: the dictator makes the allocation, and the recipient must accept this decision.

Once you have understood these instructions, please click the button below the picture to proceed to the next page.

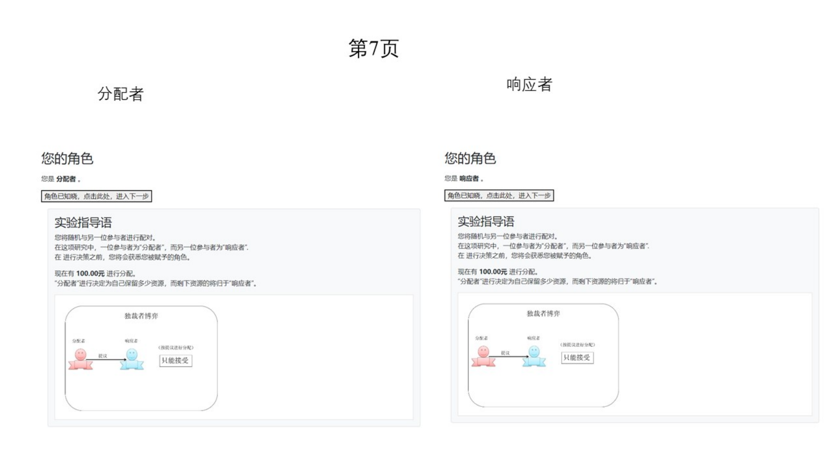

Dictator
Your role is: You are the dictator. Once the role is acknowledged, click here to proceed to the next page.

Recipient
Your role is: You are the recipient. Once the role is acknowledged, click here to proceed to the next page.

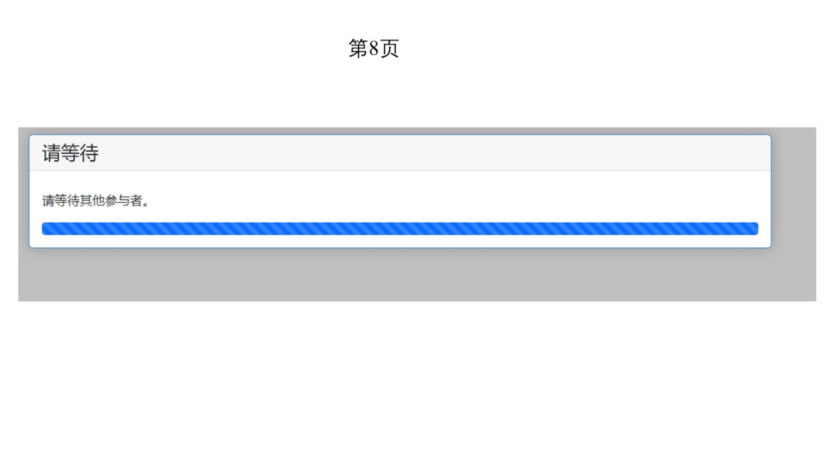

Please wait for other participants.

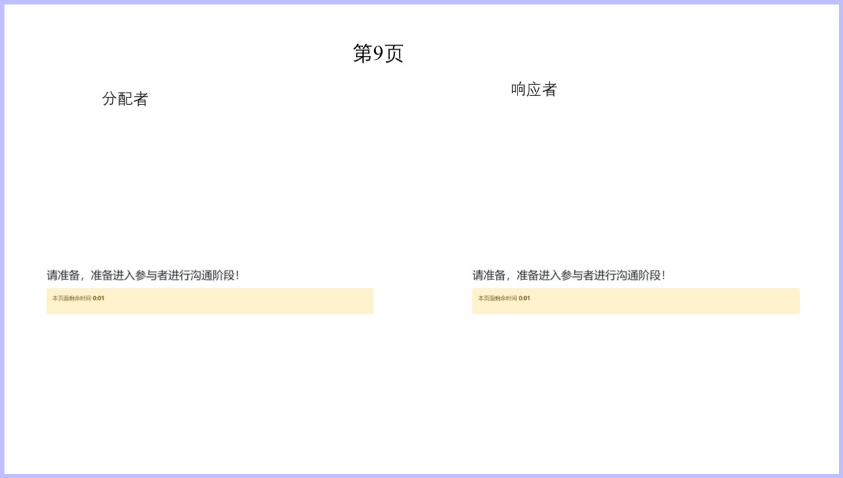

Dictator
Please ready yourself for entering the stage where you will communicate with other participants.
Time left for this page: 0.05

Recipient
Please ready yourself for entering the stage where you will communicate with other participants.
Time left for this page: 0.05

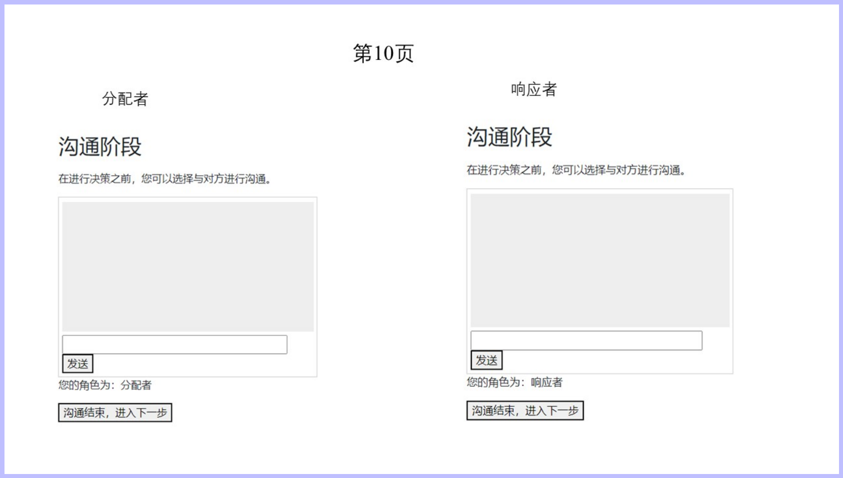

Dictator
Communication Stage:
Before making a decision, you have the option to communicate with the opponent.
Send.
Your role is: **Dictator**.
After communication is finished, proceed to the next step.

Recipient
Communication Stage:
Before making a decision, you have the option to communicate with the opponent.
Send.
Your role is: **Recipient**.
After communication is finished, proceed to the next step.

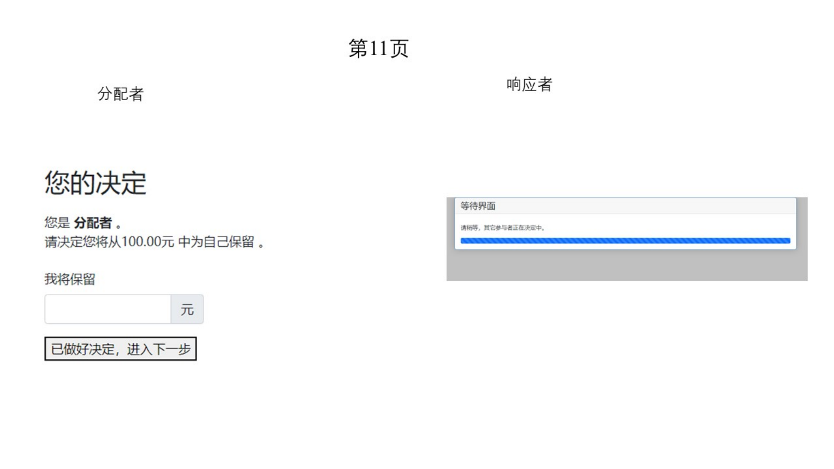

Dictator
Your Decision:
You are the dictator.
You will decide how much of the 100 RMB to keep for yourself.
I will keep:
______ RMB.
The decision has been made; proceed to the next step.

Recipient
Waiting Interface:
Please wait, other participants are making a decision.

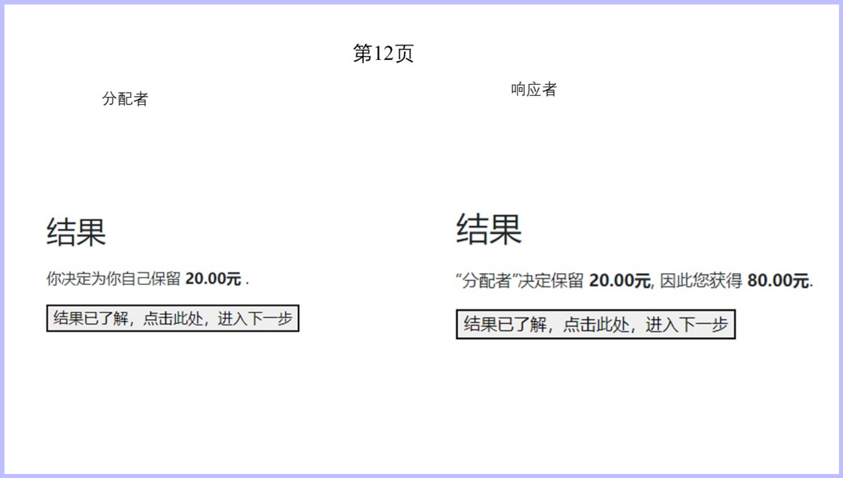

Dictator
Results:
You have decided to keep 20 RMB for yourself.
After you have understood the results, click here to proceed to the next step.

Recipient
Results:
The dictator has decided to keep 20 RMB, so you receive 80 RMB.
After you have understood the results, click here to proceed to the next step.

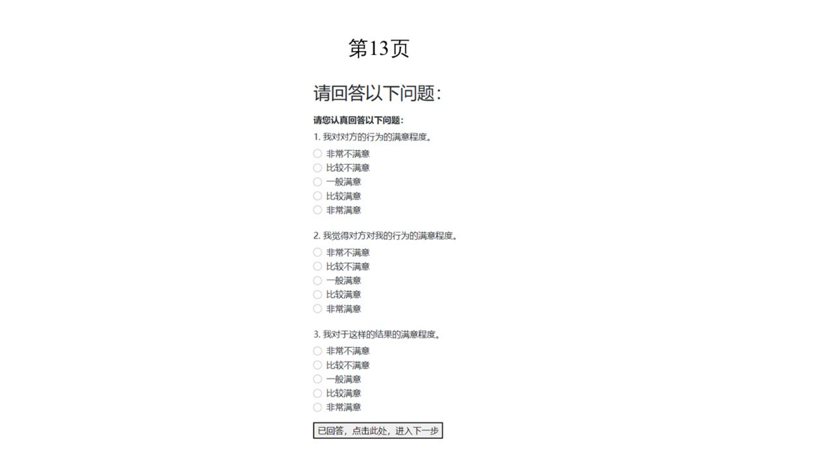
第13页

请回答以下问题:

请您认真回答以下问题:

1. 我对对方的行为的满意程度。
- 非常不满意
- 比较不满意
- 一般满意
- 比较满意
- 非常满意

2. 我觉得对方对我的行为的满意程度。
- 非常不满意
- 比较不满意
- 一般满意
- 比较满意
- 非常满意

3. 我对于这样的结果的满意程度。
- 非常不满意
- 比较不满意
- 一般满意
- 比较满意
- 非常满意

已回答，点击此处，进入下一步

Please thoughtfully answer the following questions concerning:
1. Your satisfaction with the opponent's behavior,
2. Your perception of the opponent's satisfaction with your behavior, and
3. Your satisfaction with the outcome.

For each question, you have five response options: Very Dissatisfied, Somewhat Dissatisfied, Neutral, Somewhat Satisfied, Very Satisfied.
After you have answered these questions, click here to proceed to the next step.

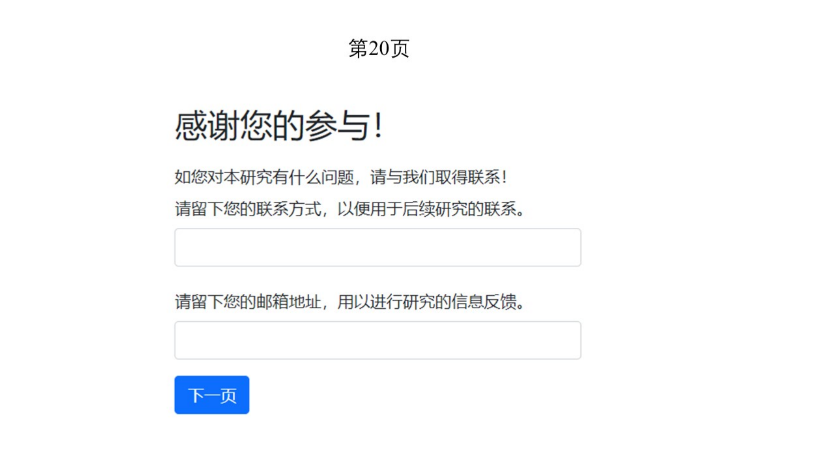

Thank you for your participation.
If you have any questions about this study, please feel free to contact us.
For future research communications, kindly leave your contact number.
Additionally, please provide your email address to receive feedback regarding the study.

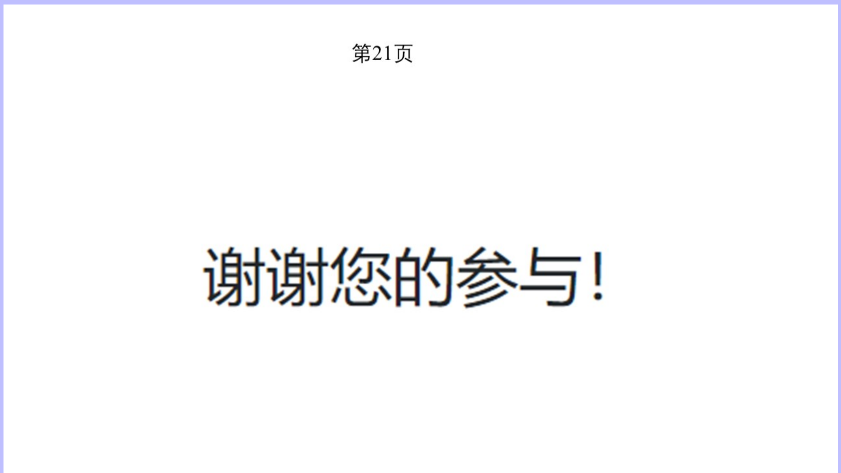

Thank you for your participants.

3 the Imagined Eye Condition

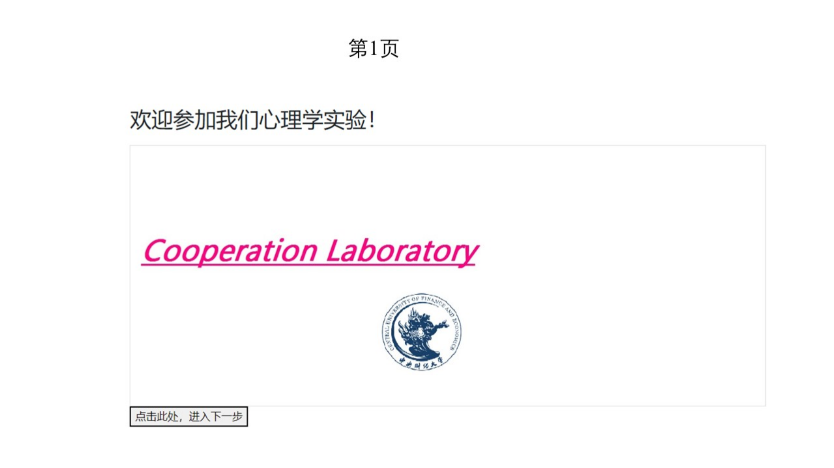

Welcome to our psychology experiments.
Cooperation Laboratory.
Click here to the next page.

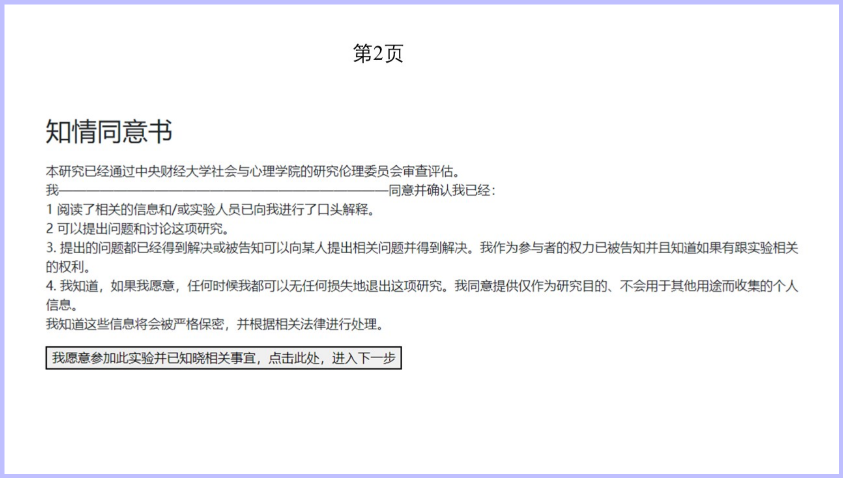

第2页

知情同意书

本研究已经通过中央财经大学社会与心理学院的研究伦理委员会审查评估。
我——————————————————————————同意并确认我已经:
1 阅读了相关的信息和/或实验人员已向我进行了口头解释。
2 可以提出问题和讨论这项研究。
3. 提出的问题都已经得到解决或被告知可以向某人提出相关问题并得到解决。我作为参与者的权力已被告知并且知道如果有跟实验相关的权利。
4. 我知道，如果我愿意，任何时候我都可以无任何损失地退出这项研究。我同意提供仅作为研究目的、不会用于其他用途而收集的个人信息。
我知道这些信息将会被严格保密，并根据相关法律进行处理。

我愿意参加此实验并已知晓相关事宜，点击此处，进入下一步

Informed Consent

This study has been reviewed and approved by the Research Ethics Committee of [anonymous for review reason]. By participating, I confirm that:

1. I have read and understood the relevant information provided about the study, or it has been explained to me verbally by the experimenter.
2. I have the opportunity to ask questions and discuss any aspect of the study.
3. Any questions I have asked have been satisfactorily addressed. I am informed that I can continue to seek clarification and ask further questions as needed. I am also aware of my rights as a participant, including any specific rights related to this experiment.
4. I understand that I am free to withdraw from this study at any time without any penalty. I consent to provide personal information for the purposes of this research, with the assurance that it will be used solely for research purposes and handled with strict confidentiality in compliance with relevant laws.

I agree to participate in this experiment and have been fully informed about its nature. To proceed to the next page, please click here.

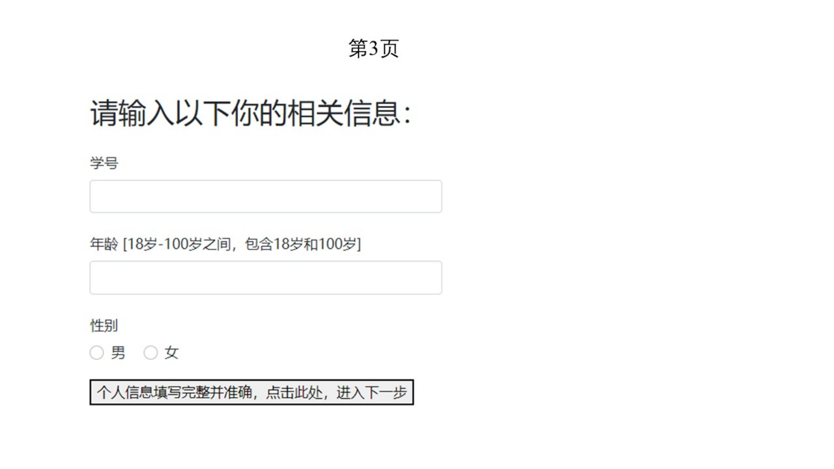

Enter your information:
student ID
Age (18-100 years, including 18 and 100 years old)
Gender: Male; Female.
I have completed my personal information. Click here to proceed to the next page.

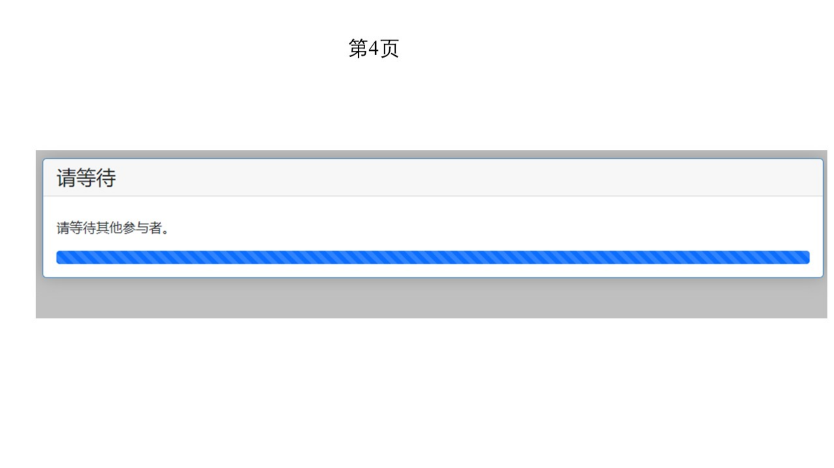

Please wait for other participants.

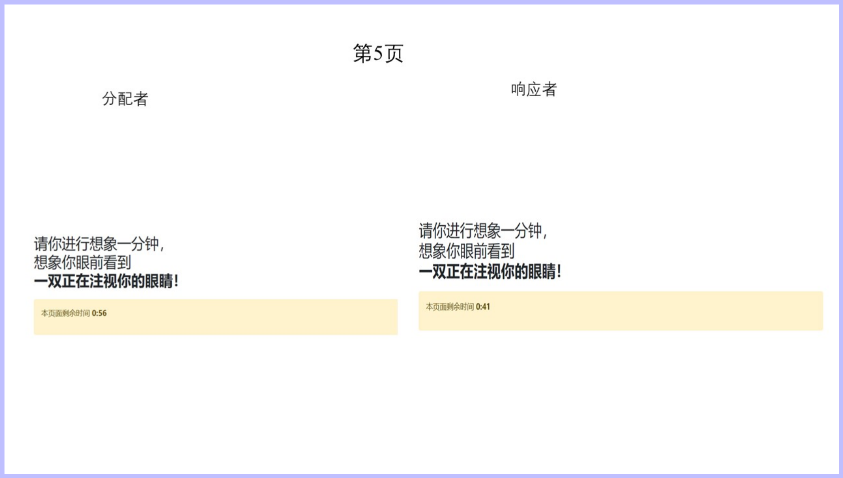

Dictator
Please imagine for a minute, imagine **a pair of eyes in front of you staring at you.**
Time left for this page: 0:56.

Recipient
Please imagine for a minute, imagine **a pair of eyes in front of you staring at you.**
Time left for this page: 0:41.

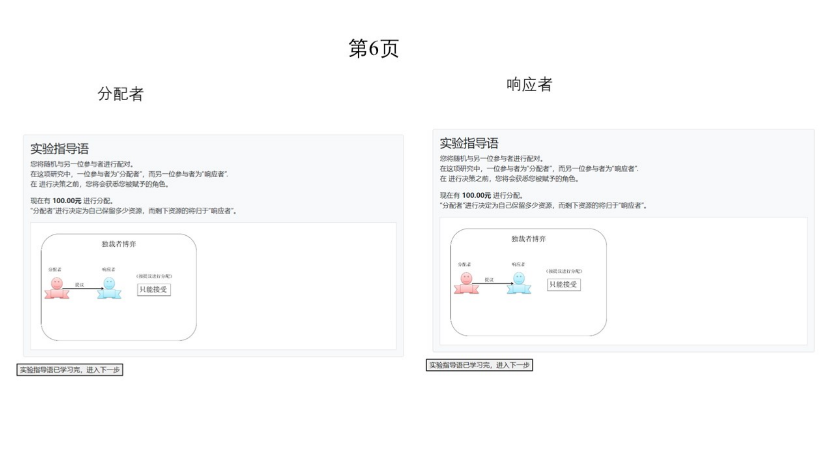

Dictator

Experiment Instructions:
You will be randomly paired with another participant for this study. In this experiment, there will be two roles: one participant will be the ”dictator”, and the other will be the ”recipient”.
Before you make any decision, your role in the study will be clearly communicated to you.
A total of **100** RMB will be available for allocation. The dictator will decide the amount of this resource they wish to keep for themselves. The remaining resources will then be allocated to the recipient.
Below, you will find an illustrative picture that briefly describes the dynamics of the Dictator Game: the dictator makes the allocation, and the recipient must accept this decision.
Once you have understood these instructions, please click the button below the picture to proceed to the next page.

Recipient

Experiment Instructions:
You will be randomly paired with another participant for this study. In this experiment, there will be two roles: one participant will be the ”dictator”, and the other will be the ”recipient”.
Before you make any decision, your role in the study will be clearly communicated to you.
A total of **100** RMB will be available for allocation. The dictator will decide the amount of this resource they wish to keep for themselves. The remaining resources will then be allocated to the recipient.
Below, you will find an illustrative picture that briefly describes the dynamics of the Dictator Game: the dictator makes the allocation, and the recipient must accept this decision.
Once you have understood these instructions, please click the button below the picture to proceed to the next page.

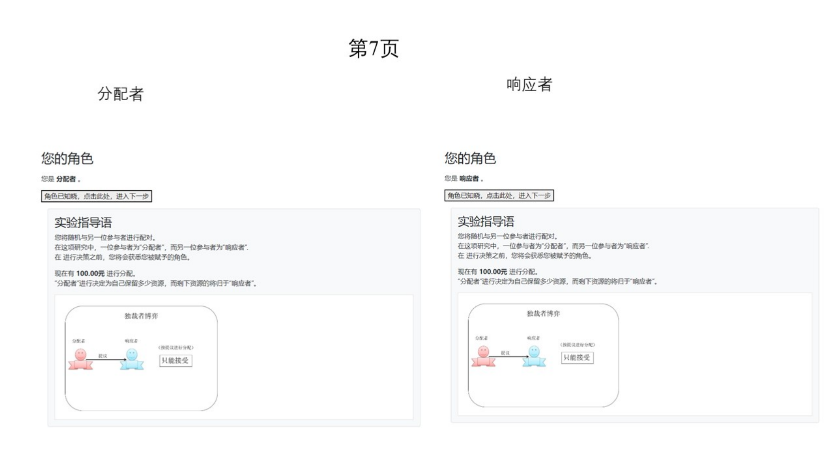

Dictator
Your role is: You are the dictator. Once the role is acknowledged, click here to proceed to the next page.

Recipient
Your role is: You are the recipient. Once the role is acknowledged, click here to proceed to the next page.

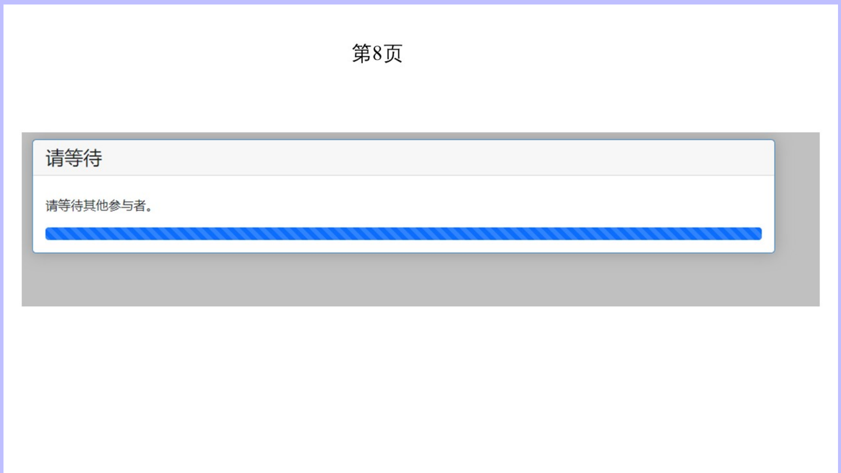

Please wait for other participants.

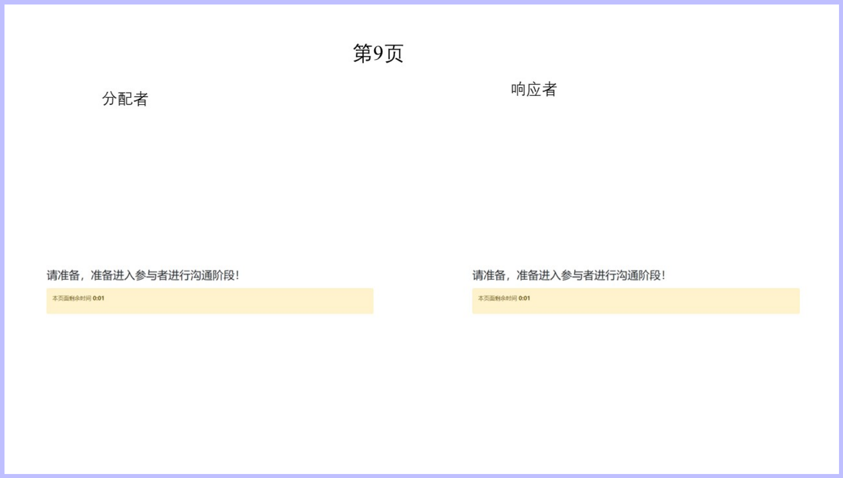

Dictator
Please ready yourself for entering the stage where you will communicate with other participants.
Time left for this page: 0.05

Recipient
Please ready yourself for entering the stage where you will communicate with other participants.
Time left for this page: 0.05

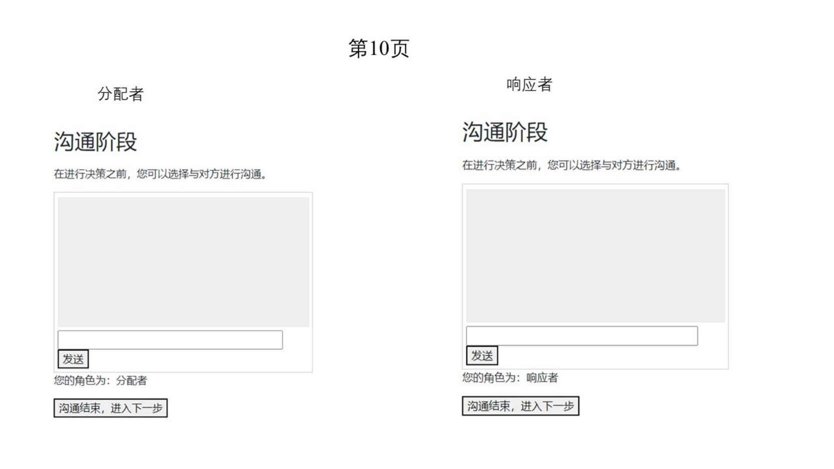

Dictator
Communication Stage:
Before making a decision, you have the option to communicate with the opponent.
Send.
Your role is: **Dictator**.
After communication is finished, proceed to the next step.

Recipient
Communication Stage:
Before making a decision, you have the option to communicate with the opponent.
Send.
Your role is: **Recipient**.
After communication is finished, proceed to the next step.

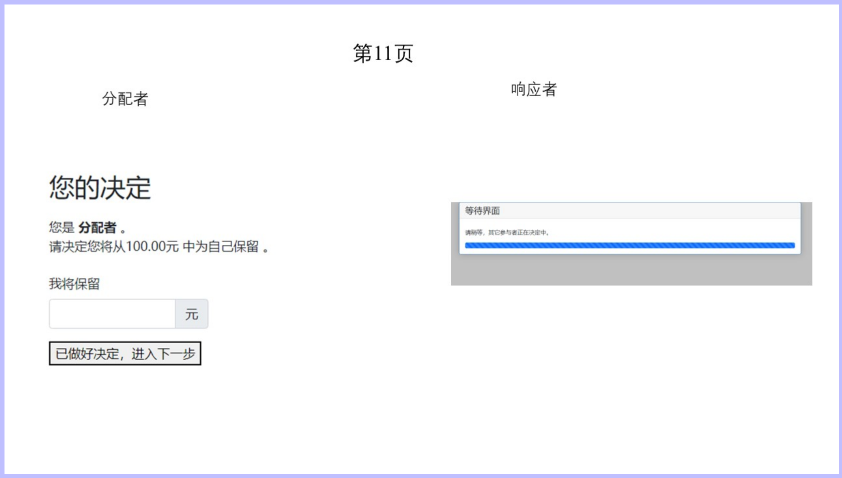

Dictator
Your Decision:
You are the dictator.
You will decide how much of the 100 RMB to keep for yourself.
I will keep:
______ RMB.
The decision has been made; proceed to the next step.

Recipient
Waiting Interface:
Please wait, other participants are making a decision.

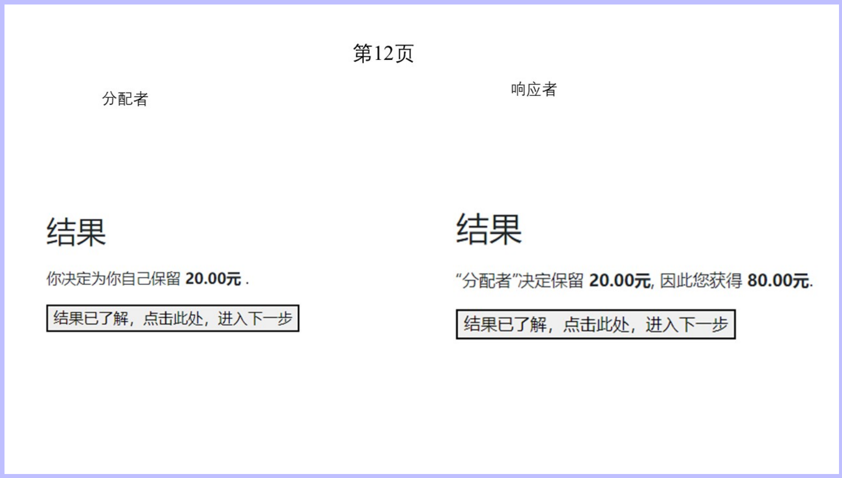

Dictator
Results:
You have decided to keep 20 RMB for yourself.
After you have understood the results, click here to proceed to the next step.

Recipient
Results:
The dictator has decided to keep 20 RMB, so you receive 80 RMB.
After you have understood the results, click here to proceed to the next step.

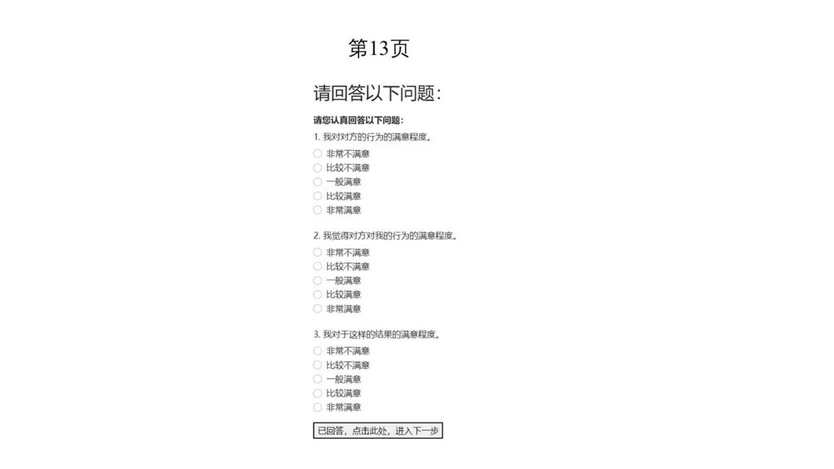

Please thoughtfully answer the following questions concerning:
1. Your satisfaction with the opponent's behavior,
2. Your perception of the opponent's satisfaction with your behavior, and
3. Your satisfaction with the outcome.
For each question, you have five response options: Very Dissatisfied, Somewhat Dissatisfied, Neutral, Somewhat Satisfied, Very Satisfied.
After you have answered these questions, click here to proceed to the next step.

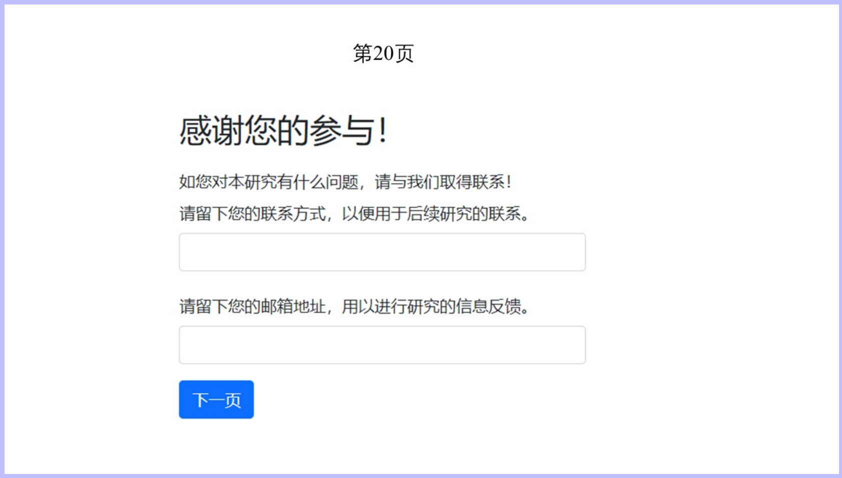
第20页

感谢您的参与！

如您对本研究有什么问题，请与我们取得联系！

请留下您的联系方式，以便用于后续研究的联系。

请留下您的邮箱地址，用以进行研究的信息反馈。

下一页

Thank you for your participation.
If you have any questions about this study, please feel free to contact us. For future research communications, kindly leave your contact number. Additionally, please provide your email address to receive feedback regarding the study.

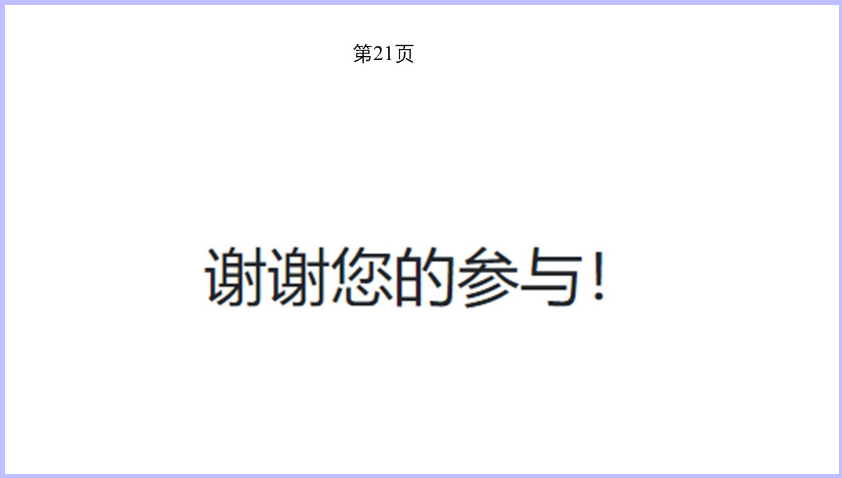
第21页

谢谢您的参与！

Thank you for your participants.

4 the Imagined Flower condition

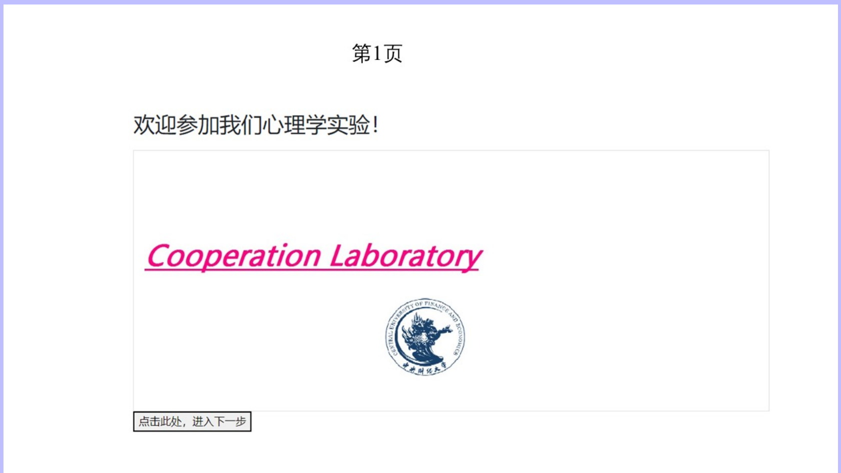

Welcome to our psychology experiments.
Cooperation Laboratory.
Click here to the next page.

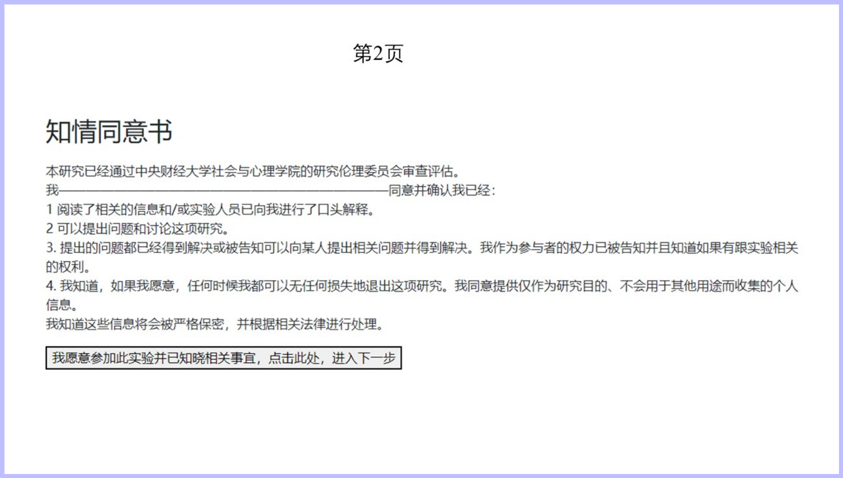

第2页

知情同意书

本研究已经通过中央财经大学社会与心理学院的研究伦理委员会审查评估。
我————————————————————————同意并确认我已经：
1 阅读了相关的信息和/或实验人员已向我进行了口头解释。
2 可以提出问题和讨论这项研究。
3. 提出的问题都已经得到解决或被告知可以向某人提出相关问题并得到解决。我作为参与者的权力已被告知并且知道如果有跟实验相关的权利。
4. 我知道，如果我愿意，任何时候我都可以无任何损失地退出这项研究。我同意提供仅作为研究目的、不会用于其他用途而收集的个人信息。
我知道这些信息将会被严格保密，并根据相关法律进行处理。

我愿意参加此实验并已知晓相关事宜，点击此处，进入下一步

Informed Consent

This study has been reviewed and approved by the Research Ethics Committee of [anonymous for review reason]. By participating, I confirm that:

1. I have read and understood the relevant information provided about the study, or it has been explained to me verbally by the experimenter.
2. I have the opportunity to ask questions and discuss any aspect of the study.
3. Any questions I have asked have been satisfactorily addressed. I am informed that I can continue to seek clarification and ask further questions as needed. I am also aware of my rights as a participant, including any specific rights related to this experiment.
4. I understand that I am free to withdraw from this study at any time without any penalty. I consent to provide personal information for the purposes of this research, with the assurance that it will be used solely for research purposes and handled with strict confidentiality in compliance with relevant laws.

I agree to participate in this experiment and have been fully informed about its nature. To proceed to the next page, please click here.

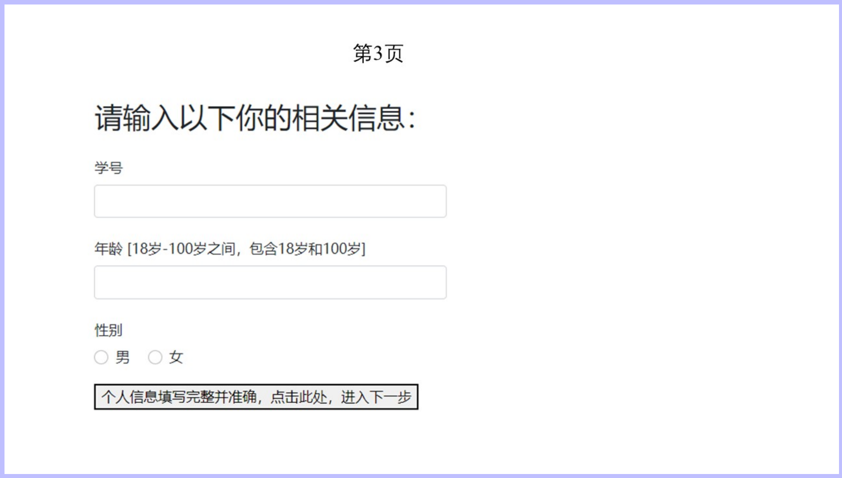

Enter your information:
student ID
Age (18-100 years, including 18 and 100 years old)
Gender: Male; Female.
I have completed my personal information. Click here to proceed to the next page.

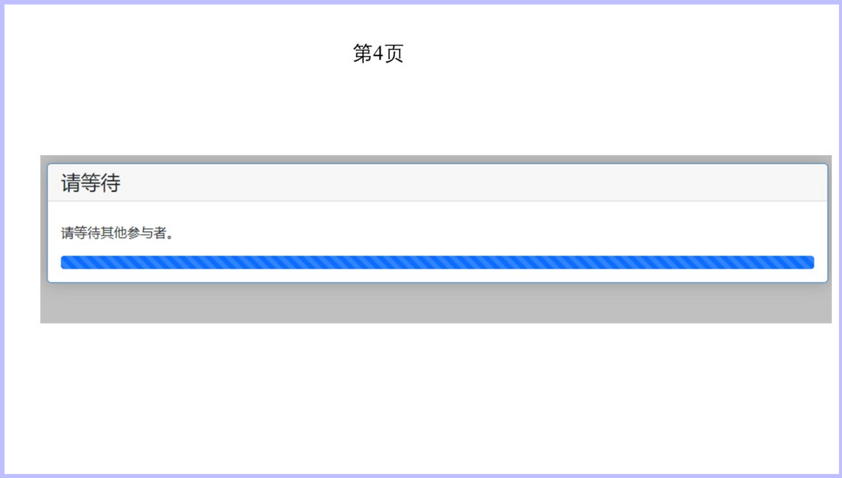

Please wait for other participants.

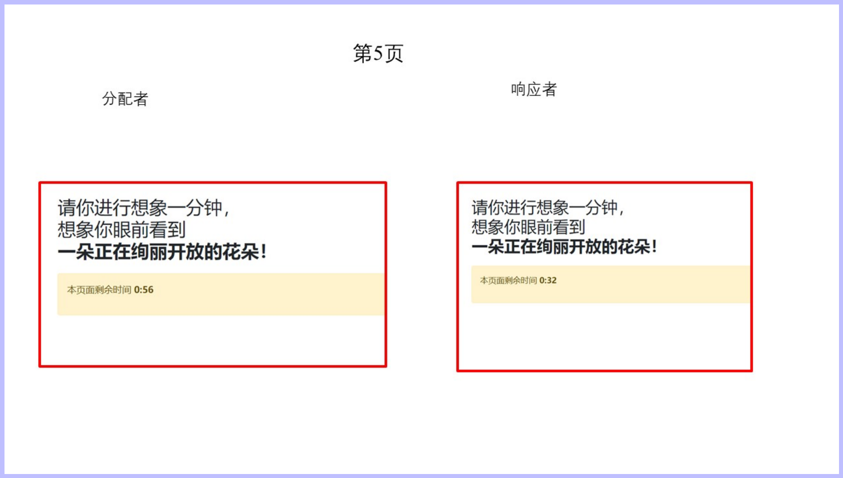

Dictator
Please imagine for a minute, imagine **seeing a flower blooming brilliantly**.
Time left for this page: 0:56.

Recipient
Please imagine for a minute, imagine **seeing a flower blooming brilliantly**.
Time left for this page: 0:32.

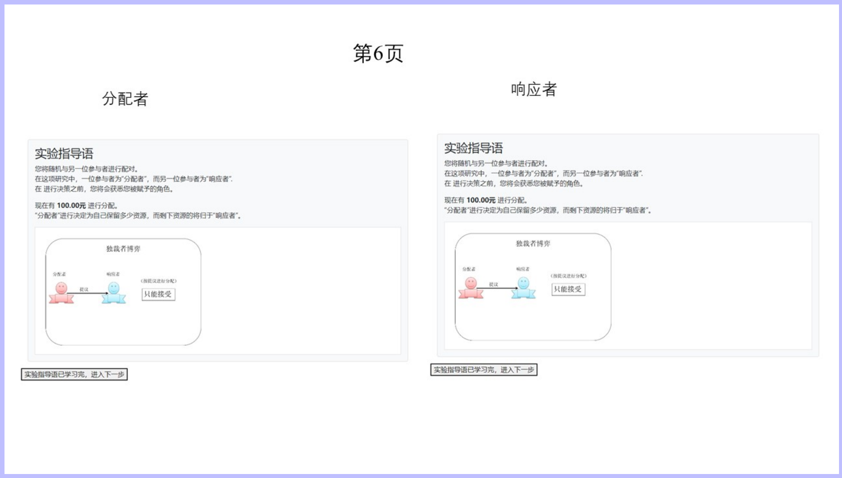

Dictator

Experiment Instructions:

You will be randomly paired with another participant for this study. In this experiment, there will be two roles: one participant will be the ”dictator”, and the other will be the ”recipient”.

Before you make any decision, your role in the study will be clearly communicated to you.

A total of **100** RMB will be available for allocation. The dictator will decide the amount of this resource they wish to keep for themselves. The remaining resources will then be allocated to the recipient.

Below, you will find an illustrative picture that briefly describes the dynamics of the Dictator Game: the dictator makes the allocation, and the recipient must accept this decision.

Once you have understood these instructions, please click the button below the picture to proceed to the next page.

Recipient

Experiment Instructions:

You will be randomly paired with another participant for this study. In this experiment, there will be two roles: one participant will be the ”dictator”, and the other will be the ”recipient”.

Before you make any decision, your role in the study will be clearly communicated to you.

A total of **100** RMB will be available for allocation. The dictator will decide the amount of this resource they wish to keep for themselves. The remaining resources will then be allocated to the recipient.

Below, you will find an illustrative picture that briefly describes the dynamics of the Dictator Game: the dictator makes the allocation, and the recipient must accept this decision.

Once you have understood these instructions, please click the button below the picture to proceed to the next page.

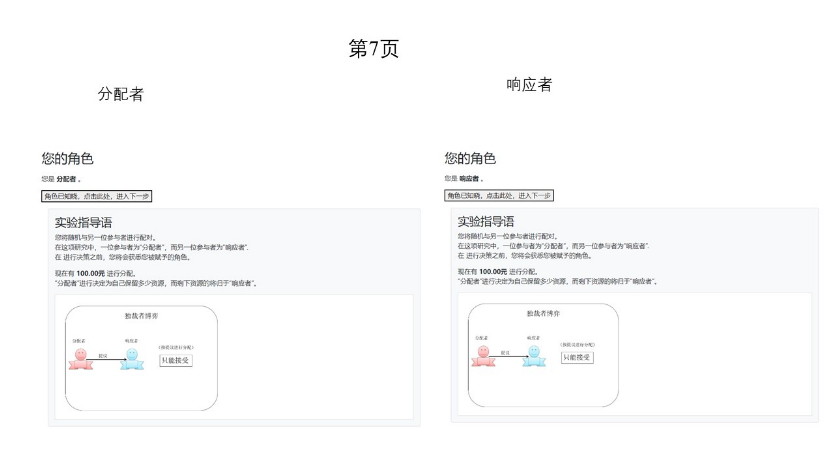

Dictator
Your role is: You are the dictator. Once the role is acknowledged, click here to proceed to the next page.

Recipient
Your role is: You are the recipient. Once the role is acknowledged, click here to proceed to the next page.

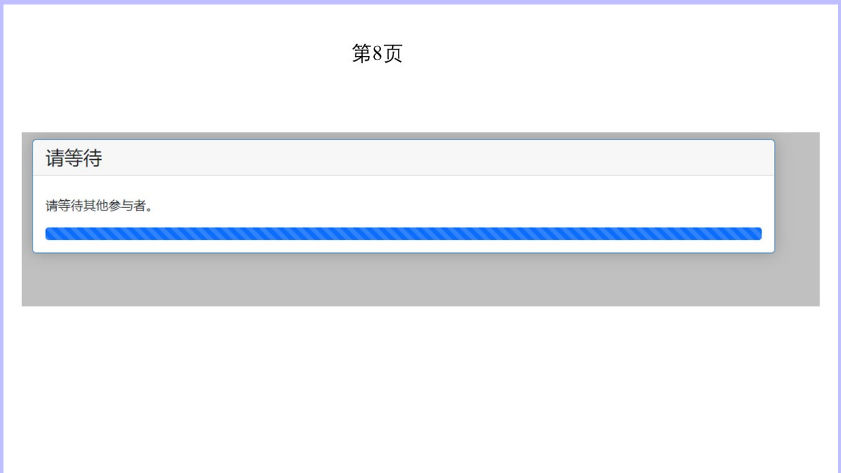

Please wait for other participants.

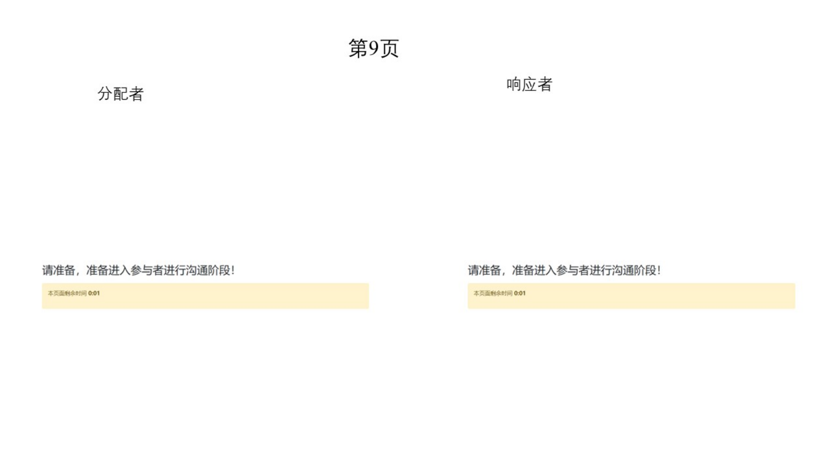

Dictator
Please ready yourself for entering the stage where you will communicate with other participants.
Time left for this page: 0.05

Recipient
Please ready yourself for entering the stage where you will communicate with other participants.
Time left for this page: 0.05

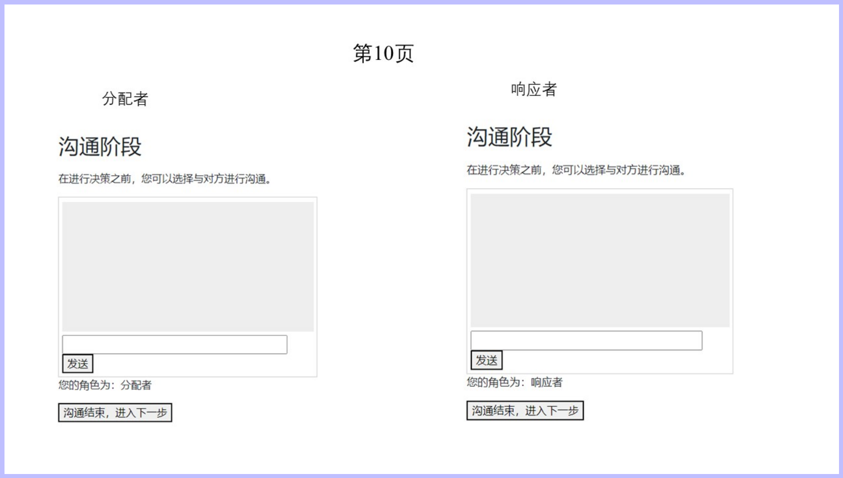

Dictator
Communication Stage:
Before making a decision, you have the option to communicate with the opponent.
Send.
Your role is: **Dictator**.
After communication is finished, proceed to the next step.

Recipient
Communication Stage:
Before making a decision, you have the option to communicate with the opponent.
Send.
Your role is: **Recipient**.
After communication is finished, proceed to the next step.

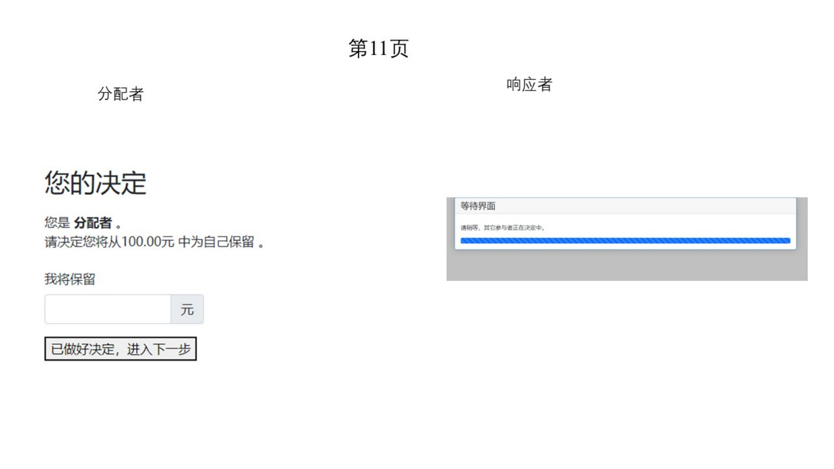

Dictator
Your Decision:
You are the dictator.
You will decide how much of the 100 RMB to keep for yourself.
I will keep:
______ RMB.
The decision has been made; proceed to the next step.

Recipient
Waiting Interface:
Please wait, other participants are making a decision.

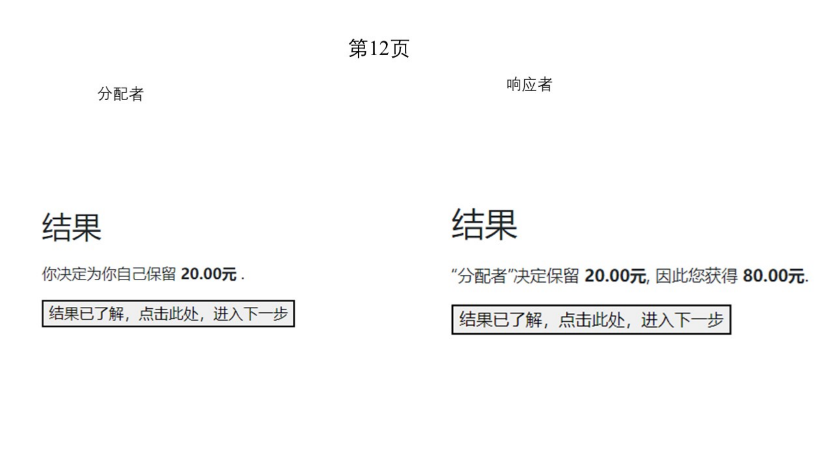

Dictator
Results:
You have decided to keep 20 RMB for yourself.
After you have understood the results, click here to proceed to the next step.

Recipient
Results:
The dictator has decided to keep 20 RMB, so you receive 80 RMB.
After you have understood the results, click here to proceed to the next step.

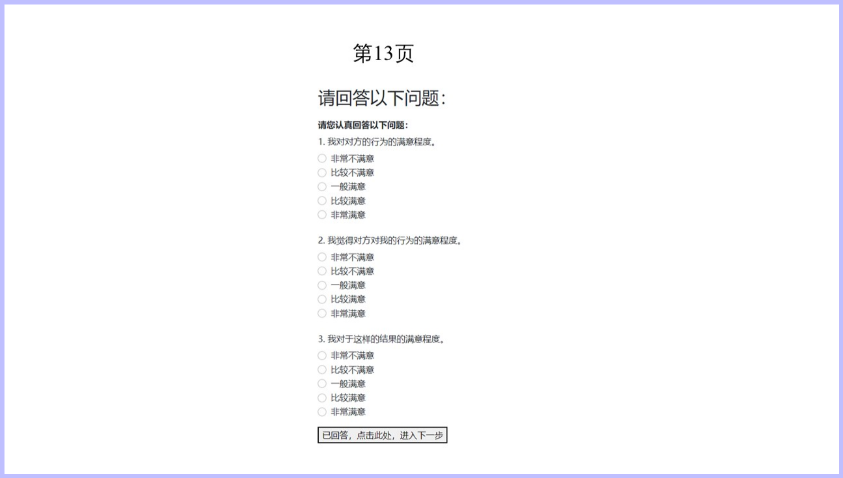

Please thoughtfully answer the following questions concerning:
1. Your satisfaction with the opponent's behavior,
2. Your perception of the opponent's satisfaction with your behavior, and
3. Your satisfaction with the outcome.
For each question, you have five response options: Very Dissatisfied, Somewhat Dissatisfied, Neutral, Somewhat Satisfied, Very Satisfied.
After you have answered these questions, click here to proceed to the next step.

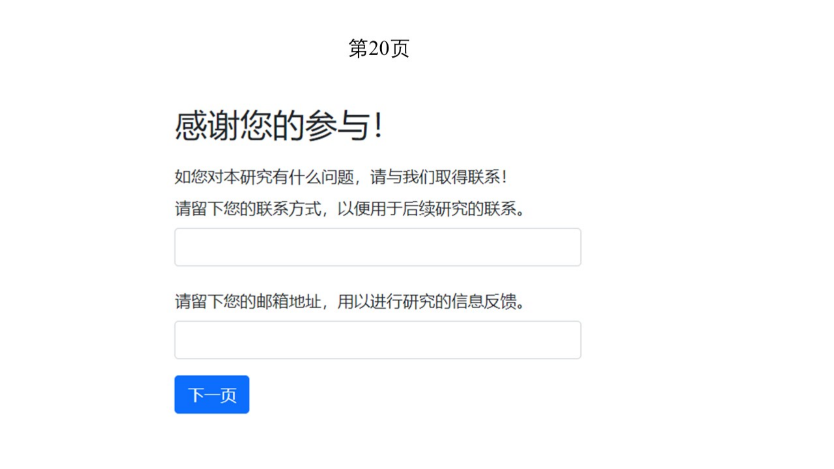

Thank you for your participation.
If you have any questions about this study, please feel free to contact us. For future research communications, kindly leave your contact number. Additionally, please provide your email address to receive feedback regarding the study.

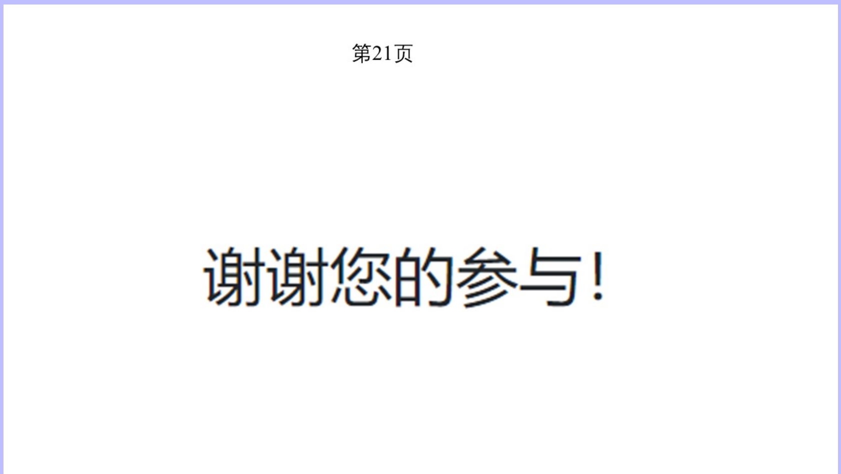

Thank you for your participants.
